# 2,4-Dichlorophenol Photodegradation by Novel Burtite-like Solid Solutions: Effect of Sn^4**+**^ and Al^3**+**^ Content on the Physicochemical Properties and Photocatalytic Performance

**DOI:** 10.1021/acsomega.6c04908

**Published:** 2026-10-01

**Authors:** Federico Manuel Reyes-Cruz, Manuel Sánchez-Cantú, Clara Tzompantzi-Flores, Irving I. Ruiz-López, Angela-Gabriela Romero-Villegas, Francisco Tzompantzi

**Affiliations:** † Facultad de Ingeniería Química, 3972 Benemérita Universidad Autónoma de Puebla, Avenida San Claudio y 18 Sur, Puebla, Puebla C.P. 72570, México; ‡ Departamento de Química, Área de Catálisis, Universidad Autónoma Metropolitana Iztapalapa, Av. San Rafael Atlixco No. 189, Iztapalapa, México City 09340, México

## Abstract

Nowadays, tailoring the physicochemical properties of novel photocatalysts is crucial for advanced environmental remediation technologies. Thus, in this work, novel solid solutions of burtite-like compounds with nominal Sn amounts of 5.3–27.8 mol % were synthesized. Materials were prepared by the co-precipitation method followed by a hydrothermal treatment. A material without tin was also prepared as a control, and all materials were characterized by X-ray powder diffraction, scanning electron microscopy, nitrogen adsorption–desorption at −196 °C, diffuse reflectance spectroscopy, and X-ray photoelectron spectroscopy. To verify their photocatalytic behavior, all samples were tested for the photocatalytic degradation of 2,4-dichlorophenol (a very toxic and recalcitrant pesticide) as a probe molecule. Aeroxide TiO_2_ P25 was also evaluated by means of comparison under the same experimental conditions. Results revealed that the amount of tin defined the crystalline phases found in the samples since compounds with low tin amounts (below 10%) produced mixtures of hydrocalumite, katoite, and burtite. On the other side, higher tin contents produced pure burtite-like phases. The obtained solid solutions were verified by cell parameter calculations and the absence of secondary crystalline phases. A new acicular morphology of burtite was obtained and assigned to the aluminum-rich solid solution. Photocatalytic experiments demonstrated that all synthesized materials were active under UV irradiation; however, the highest activity was achieved by the katoite/burtite composite (Al-B2), reaching an apparent DiPhe degradation of 54% after 30 min (estimated from UV–vis measurements), while TiO_2_ exhibited a degradation of 25%. Additionally, 75.5% mineralization was achieved after 2 h, while only 53.8% was attained with TiO_2_. The most active material was evaluated over six consecutive cycles, with photocatalytic activity dropping from 70% to 25% mineralization between the first and sixth runs due to a lixiviation process. Results demonstrated that burtite’s structure can be modified and its physicochemical characteristics can be adjusted to produce potential photocatalytic materials.

## Graphical abstract

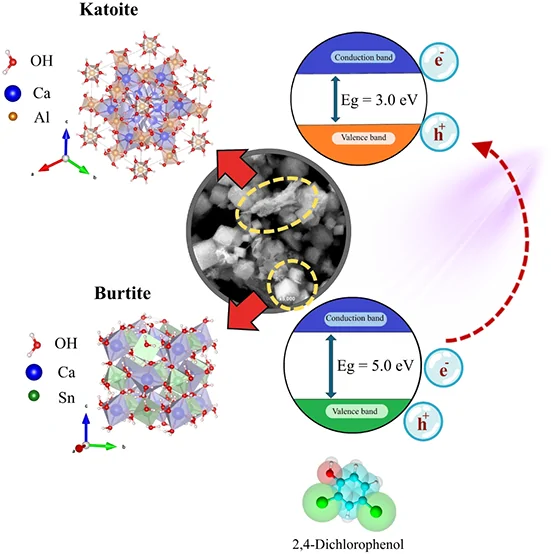

## 1 Introduction

Burtite, a calcium hydroxystannate CaSn­(OH)_6_, belongs to a group of materials that includes schoenfliesite, MgSn­(OH)_6_; wickmanite, MnSn­(OH)_6_, natanite, FeSn­(OH)_6_, vismirnovite, ZnSn­(OH)_6_, and mushistonite, CuSn­(OH)_6_, among others.^1^

CaSn­(OH)_6_ can be represented as “double-ReO_3_” structure, where Ca^2+^ and Sn^4+^ are alternately distributed to form the octahedral chains.^2^ In this sense, cations and anions are arranged in the form of a cubic unit cell, belonging to the space group *Pn*3̅*m*, where the calcium ions are present at the corners, while tin ions are located between them.^3^

Because of its unique physicochemical characteristics (crystalline structure, morphologies, hydroxyl abundance, among others), several synthesis methods have been developed for its production. Among these, coprecipitation is the most employed;^1,2,4^ however, other methods such as hydrothermal,^5,6^ microwave,^7^ and sonochemical precipitation^8,9^ have been reported.

Although burtite has been employed in flame-retardant formulations,^4,10,11^ catalysts,^11^ and fuel additives;^3^ its use as a catalyst, catalyst support, and/or catalyst precursor in the photocatalysis field is scarce.

Table 1 provides a comparative overview of the photocatalytic systems reported for burtite and modified burtite-like compounds. Studies have focused on the degradation of some model organic dyes and organic contaminants.

**Table 1:** Summary of Burtite Condition Reported in the Literature for Heterogeneous Photocatalytic Process

Year	Material	Light irradiation	Photocatalytic system	Time, min	Target pollutant	Photocatalytic performance, %
2011 ^9^	CaSn(OH)_6_	UV (254 nm)	Gas-phase	60	Benzene	20
2011 ^9^	CaSn(OH)_6_	UV (254 nm)	Liquid-phase	100	Methyl orange dye	98.7
2014 ^12^	CaSn(OH)_6_	UV	Liquid-phase	55	Methylene blue dye	∼15
2014 ^12^	Pd/CaSn(OH)_6_ nanocomposite	UV	Liquid-phase	55	Methylene blue dye	Above 95
2017 ^6^	CaSn(OH)_6_/SnO_2_	UV cutoff filter (λ > 420 nm)	Liquid-phase	15	Methyl blue dye	∼80
2017 ^6^	CaSn(OH)_6_/SnO_2_ doped with Fe^3+^	UV cutoff filter (λ > 420 nm)	Liquid-phase	15	Methyl blue dye	∼90
2017 ^6^	CaSn(OH)_6_/SnO_2_	Visible	Liquid-phase	300	Methyl blue dye	Above 15
2017 ^6^	CaSn(OH)_6_/SnO_2_ doped with Fe^3+^	Visible	Liquid-phase	300	Methyl blue dye	∼80
2017 ^13^	CaSn(OH)_6_	Visible (554 nm)	Liquid-phase	30	Tetracycline	17.4
2017 ^13^	Ag@AgCl nanoparticle-modified CaSn(OH)_6_	Visible (554 nm)	Liquid-phase	30	Tetracycline	94.2
2017 ^13^	CaSn(OH)_6_	Visible (554 nm)	Liquid-phase	10	Rhodamine B	Below 10
2017 ^13^	Ag@AgCl nanoparticle-modified CaSn(OH)_6_	Visible (554 nm)	Liquid-phase	10	Rhodamine B	97
2017 ^13^	CaSn(OH)_6_	Visible (554 nm)	Liquid-phase	25	Crystal violet	Above 95
2017 ^13^	Ag@AgCl nanoparticle-modified CaSn(OH)_6_	Visible (554 nm)	Liquid-phase	25	Crystal violet	∼97
2017 ^13^	CaSn(OH)_6_	Visible (554 nm)	Liquid-phase	60	Bisphenol A propane	Below 10
2017 ^13^	Ag@AgCl nanoparticle-modified CaSn(OH)_6_	Visible (554 nm)	Liquid-phase	60	Bisphenol A propane	∼80
2018 ^7^	CaSn(OH)_6_	UV	Liquid-phase	450	Methyl blue dye	Above 70
2018 ^7^	CaSn(OH)_6_-CTAB surfactant	UV	Liquid-phase	450	Methyl blue dye	Above 90
2020 ^14^	Sn self-doped CaSn(OH)_6_	UV (365 nm)	Gas-phase	60	Formaldehyde	79
2025 ^15^	NH_2_–UiO-66/CaSn(OH)_6_/SnO_2_ composite material	Visible (420 nm)	Gas-phase	30	NO	64.4

Notably, applications of burtite are not only scarce but also focused on modifying its structure. In this sense, only two examples have been found in the literature. In the first case, Dong *et al.* prepared boron-doped burtite for its use to obtain flame-retardant PVC composites,^4^ while Liu *et al.* prepared Fe^3+^-doped compounds for their evaluation as photocatalysts.^6^ In both cases, the shift toward higher or lower values of two-theta was assigned to lattice contraction or expansion, indicating boron or iron incorporation, respectively. Specifically, since aluminum possesses a distinct oxidation state and a smaller ionic radius than calcium or tin, its incorporation into the burtite structure is expected to modify its structural, physicochemical, and photocatalytic properties.^16–18^

To our knowledge, neither solid solution of aluminum-containing burtite nor its evaluation as a photocatalyst has been reported previously. Thus, in this work, a series of aluminum, calcium, and tin compounds was prepared by coprecipitation followed by a hydrothermal treatment, maintaining the Ca:Al molar ratio and varying the tin content in the 5.3–27.8 mol % range. 2,4-ichlorophenol (DiPhe) was chosen as a probe molecule given its toxic effects not only on human beings but also on the environment.

## 2 Materials and Methods

### 2.1 Chemicals

Ca­(NO_3_)_2_·4H_2_O (99%, Reasol), Al­(NO_3_)_3_·9H_2_O (98%, Meyer), SnCl_4_·5H_2_O (99%, J.T.Baker), NH_4_NO_3_ (95%, Meyer), KOH (85%, Meyer), HNO_3_ (64%, Fermont) and 2,4-ichlorophenol (99%, Aldrich), Aeroxide TiO_2_ P25 (99%, Evonik) were used as received. Bidistilled water was supplied by a local water purification plant.

### 2.2 Samples’ Preparation

Materials with and without tin were synthesized by coprecipitation, with some modifications to the method reported earlier.^19^ The specific conditions for each material preparation are provided in the Supporting Information section. Materials were labeled as Al-B0, Al-B1, Al-B2, Al-B3, and Al-B4, where 0 = 0, 1 = 5.3, 2 =10.3, 3 =19.5 and 4 = 27.8 mol % of tin, respectively.

### 2.3 Materials Characterization

All compounds were analyzed by X-ray powder diffraction (XRD) on a Bruker D2 Phaser diffractometer with Cu Kα radiation. The diffractograms were acquired over 2*θ* = 5–70° with a step size of 0.04° and a counting time of 0.5 s per point. The JCPDS (Joint Committee on Powder Diffraction Standards) database was used for identification. The characteristic vibrational bands of the materials were analyzed by Fourier transform infrared spectroscopy (FTIR) using a Shimadzu Affinity-1 spectrophotometer equipped with a total-reflectance attenuated accessory with a ZnSe crystal window. Transmittance was reported as a function of wavenumber between 500 and 4000 cm^–1^. Diffuse reflectance spectroscopy (DRS) was used to study the materials’ optical properties. UV–vis diffuse reflectance spectra were measured on a Varian Cary-100 spectrophotometer equipped with an integrating sphere accessory, using BaSO_4_ as the reference, in the wavelength range from 200 to 600 nm. The band gap energy (*E*_g_) was determined from values obtained using the Kubelka–Munk equation (see eq 1) and the Tauc plot derived from eq 2, by extrapolating the linear portion of the (*F*(*R*) × *h*υ)^2^ vs *h*υ curves to *F*(*R*) = 0. In these equations, *R* is the reflectance value, *h* is the Planck constant, υ is the relation between the speed of light and the wavelength of the incident photons (υ = *c*·λ^–1^), and *A* is a proportionality constant independent of the photon energy and equal to one.

1 $$F\left(R\right)=\frac{{\left(1-R\right)}^{2}}{2R}$$

2 $${\left(F\left(R\right)\times h\upsilon \right)}^{2}=A\left(h\upsilon -E_{g}\right)$$

Photoluminescence analysis was carried out on a fluorescence spectrophotometer FS-2 with an excitation wavelength (λ_ex_) of 254 nm, over the 400–900 nm range. X-ray photoelectron spectroscopy (XPS) was recorded using a Thermo Fisher Scientific K-Alpha X-ray photoelectron spectrometer equipped with an Al Kα monochromatic X-ray source (1486.6 eV) and a 400 μm spot size. For data analysis, the regions were calibrated considering C 1s at 284.6 eV. The elemental and morphological analyses were performed using scanning electron microscopyon a JEOL JSM-7600f microscope operated at 20 kV and equipped with dispersive X-ray spectroscopy. Images were acquired from secondary electron signals at 5000×. Textural analysis, i.e., specific surface area, average pore size, and total pore volume, was determined using a Quantachrome Autosorb-3B instrument. The samples were degassed previously at 100 °C for 24 h.

### 2.4 Photocatalytic Degradation of 2,4-Dichlorophenol

Similar to other studies, photocatalytic activity was assessed in a glass batch reactor coupled with a cooling recirculation system, maintaining the reactions at room temperature.^20^ The light source was a UVP Pen-Ray Power model 11SC-1 (254 nm, 4.4 mW/cm^2^; Analytik Jena Co., Upland, CA, USA) protected by a quartz tube. Experiments were conducted without UV filters. Into the reactor, 200 mL of 2,4-dichlorophenol (DiPhe) at 40 ppm and 200 mg of catalyst were added, maintaining air bubbling and magnetic stirring at 1 mL/s and 800 rpm, respectively.

The experiments were conducted as follows. First, photolysis was carried out to determine DiPhe degradation under UV radiation; an experiment performed in the dark for 1 h was conducted to reach adsorption–desorption equilibrium conditions. Then, photocatalytic activity was monitored throughout the time. The solution was irradiated with UV light for 6 h, and aliquots (3 mL) were collected every 15 min for 3 h; thereafter, every hour until the end of the experiment. Because the suspension was continuously stirred, both the liquid phase and the suspended photocatalyst were removed proportionally, maintaining an approximately constant catalyst concentration in the remaining suspension. Nevertheless, the cumulative reduction in the reaction volume may slightly modify the irradiation geometry and photon flux per unit volume. Therefore, the results were interpreted comparatively since identical sampling and irradiation conditions were applied to all synthesized materials and the TiO_2_ reference.

Additionally, commercial Aeroxide TiO_2_ P25 was used as the reference photocatalyst. TiO_2_ was evaluated using the same catalyst loading, DiPhe concentration, solution volume, adsorption–desorption equilibrium time, irradiation source (254 nm, 4.4 mW cm^–2^), reaction time, sampling intervals, and post-treatment procedure as those employed for the synthesized materials, allowing a direct comparison of their photocatalytic performance.

The photocatalyst recovery was carried out using a membrane coupled to a PTFE (polytetrafluoroethylene) syringe filter, and the resulting solution was monitored using a Varian Cary-100 UV-Vis spectrometer (Agilent Technologies, Santa Clara, CA, USA) over the 190–350 nm range. The photocatalysis efficiency was calculated with eq 3, where *C*_0_ is the initial concentration of DiPhe and *C* is the concentration of DiPhe at a given time.^21^ Additionally, the mineralization percentage was quantified by total organic carbon (TOC) determination on a Shimadzu TOCV CSH/CSNanalyzer (Kyoto, Japan).

3 $$\eta =\frac{C_{0}-C}{C_{0}}$$

## 3 Results and Discussion

### 3.1 X-ray Diffraction

The diffractograms of the as-synthesized samples are exhibited in Figure 1. For better visualization and comparison, they were normalized. Al-B0 (sample without tin) consisted of a mixture of nitrate/carbonate hydrocalumite-like compounds, katoite (JCPDS #84-1353), and calcite (JCPDS #88-1807), which is in good agreement with previous works.^19,22^ Hydrocalumite, [Ca_2_Al­(OH)_6_]­Cl·2H_2_O, belongs to the anionic clay family (also known as layered double hydroxides ). The layered structure comprises the periodical stacking of positively charged [(Ca^2+^, Al^3+^)­(OH)_6_] octahedral layers related to brucite and negatively charged interlayers consisting of anions and water molecules. Katoite (also known as hydrogarnet), Ca_3_Al_2_(OH_12_), is a calcium aluminate with no known anion-exchange properties. Its structure consists of alternating tetrahedra and octahedra, which share corners to form a three-dimensional network. The tetrahedra are composed of four hydroxyls, H_4_O_4_ ^4^–. Octahedral sites are occupied by aluminum in 6-fold coordination. The cavities formed between the corner-sharing tetrahedra and octahedra contain calcium in 8-fold coordination.^23^

**Figure 1:**
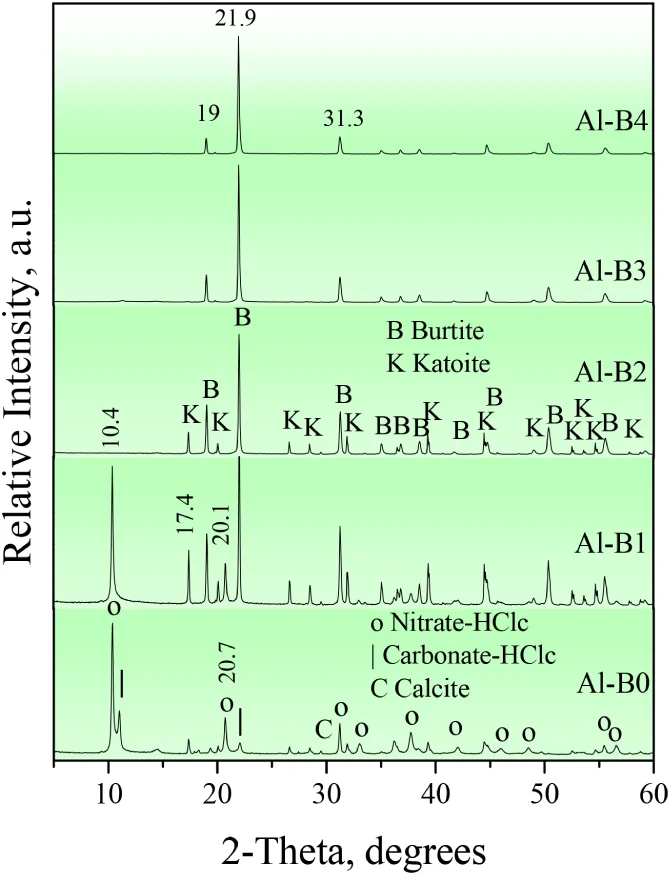
X-ray powder patterns of the synthesized samples.

Interestingly, with a tin amount as low as 5.3 mol % (Al-B1), the characteristic reflections of burtite (JCPDS #09-0030) were identified, which increased progressively until only the burtite’s phase was observed in samples Al-B3 and Al-B4. It is worth mentioning that calcite was identified in Al-B0, Al-B1, and Al-B2, but not in Al-B3 and Al-B4. This indicates that tin presence not only favors burtite’s phase but also inhibits the crystallization of secondary phases.

Cell parameters’ analysis is presented in Table 2. For hydrocalumite, *a* and *c* were calculated assuming a 3R stacking sequence, with *a* = 2*d*_(*110*)_ and *c* = 3*d*_(*003*)_, respectively. On the other hand, for burtite and katoite (both crystallizing in the cubic system), the *a* parameter was determined as 2*d*_(200)_ and *a* = 4*d*_(400)_, respectively. For Al-B0, the obtained *a* parameter value, assigned to the average cation–cation distance inside the brucite-like layers, corresponded to that reported in the literature for a Ca^2+^/Al^3+^ molar ratio of 2.^24^ In this sense, the cell parameter *c,* calculated from both reflections centered at ∼10.39 and 10.99 of 2-theta, indicated the crystallization of nitrate (JCPDS # 89-6723) and carbonate hydrocalumite-like compounds, which are attributed to nitrate ions from the metallic salts and atmospheric CO_2_. Although a hydrocalumite-like phase was still observed in the Al-B1 sample, the *a* parameter decreased from 5.731 to 5.719 Å, indicating a lattice contraction attributed to a calcium deficiency, which is in good agreement with burtite’s crystallization. Regarding katoite, which is also found during hydrocalumite’s synthesis, it also disclosed a lattice contraction (from 12.540 Å for Al-B0 to 12.518 Å for Al-B1), assigned also to a calcium deficit.

**Table 2:** Cell Parameters of the Indicated Samples

Sample	*a*, Å^a^	*a*, Å^b^	*a*, Å^c^	*c*_1_, Å^c^	*c*_2_, Å^c^
Al-B0	-	12.540	5.731	17.054	16.104
Al-B1	8.072	12.518	5.719	17.022	-
Al-B2	8.088	12.550	-	-	-
Al-B3	8.089	-	-	-	-
Al-B4	8.091	-	-	-	-

a Burtite.

b Katoite.

c Hydrocalumite.

Regarding burtite, the calculated cell parameters ranged from 8.072 to 8.091 Å, increasing gradually as the tin content increased. Nevertheless, the reported value for well-crystallized burtite (8.128 Å) was not achieved. Although the stoichiometric ratio of calcium and tin ions in burtite is 1:1, whereas the material with the highest nominal tin amount prepared in this work (Al-B4) is 1.72; the results herein presented can be assigned to a solid solution formation between calcium, aluminum, and tin ions, since the incorporation of aluminum in the lattice caused lower cell values than the expected for pure burtite, which is in good agreement with the fact that no crystalline phases attributable to calcium, aluminum, or tin were observed. Moreover, as tin increased, the aluminum-containing crystalline phases, such as hydrocalumite and katoite, gradually disappeared.

### 3.2 Fourier Transform Infrared Spectroscopy

The infrared spectra of the synthesized materials are shown and discussed in detail in the Supporting Information section. FTIR results showed, for Al-B0, the characteristic vibrations of hydrocalumite; for Al-B1, the simultaneous presence of hydrocalumite and katoite; and, finally, for Al-B2, Al-B3, and Al-B4, the characteristic vibrations of burtite were observed. The results are in good agreement with those reported by other authors.^25–27^

### 3.3 Textural Analysis

The textural properties of the synthesized materials are presented in detail in Table 3, where key parameters such as specific surface area (SSA), total pore volume, and average pore diameter are listed. The SSA value of Al-B0 (7 m^2^g^–1^) is in accordance with previous literature reports,^28^ while the remaining samples (Al-B1 to Al-B3), in which tin was incorporated, exhibited similar SSA values to Al-B0; interestingly, Al-B4 showed the highest SSA (14 m^2^ g^–1^). As will be further shown, this effect is due to the high amounts of acicular crystals observed in Al-B4, which increased the SSA value. Regarding average pore diameter, all five samples fall within the mesoporous range, with pore volumes between 0.012 and 0.016 cm^3^/g. In this regard, Al-B4 presented the highest total pore volume and pore diameter, which could also explain the difference in SSA values compared to the other samples.

**Table 3:** Textural Properties of Synthesized Samples

Sample	Specific surface area (m^2^ g^–1^)	Total pore volume (cm^3^g^–1^)	Average pore diameter (nm)
Al-B0	7	0.016	9
Al-B1	9	0.016	7.0
Al-B2	6	0.024	16
Al-B3	7	0.031	18
Al-B4	14	0.191	57

### 3.4 Morphological and Elemental Analysis

Figure 2 presents the SEM micrographs of the samples. Al-B0 showed aggregates of hexagonal-shaped particles (Figure 2), as well as small cubic particles, which are in good agreement with the reported crystalline habit of hydrocalumite^19^ and calcite.^29^ On the other side, in Al-B1, besides the compounds identified in Al-B0, cubic and small platy-like particles were also observed (see Figure 2B), which were assigned to burtite^9,30^ and katoite,^31^ correspondingly. Regarding Al-B2 (Figure 2C), only the cubic and platy-like particles were noticed.

**Figure 2:**
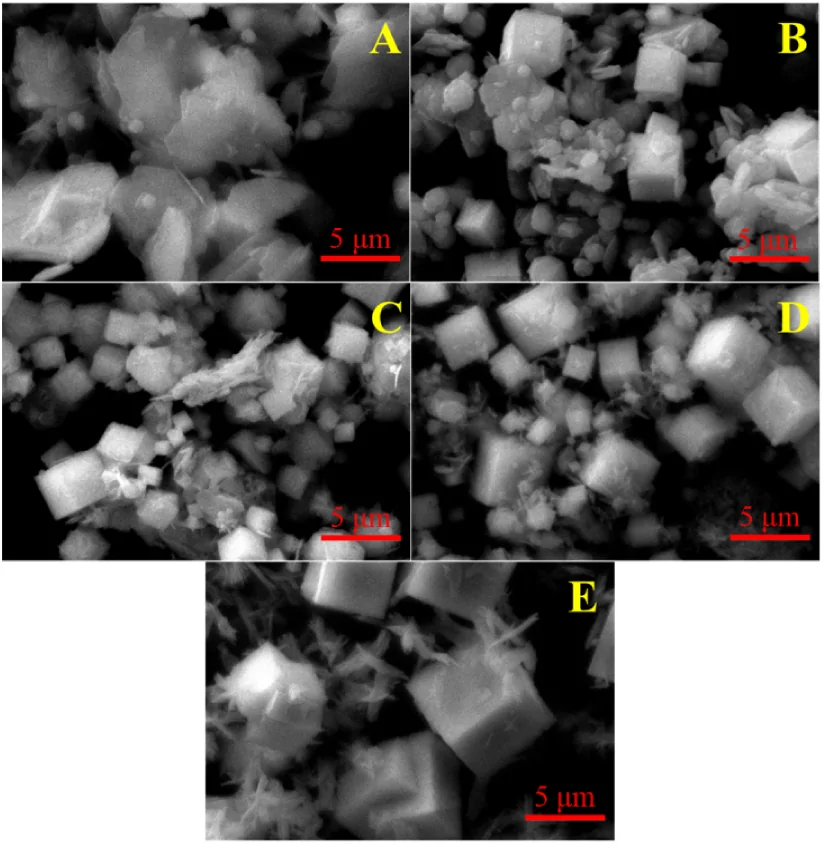
SEM micrographs: (A) Al-B0, (B) Al-B1, (C) Al-B2, (D) Al-B3, and (E) Al-B4.

It is worthwhile mentioning that Al-B3 and Al-B4 (Figure 2D and E) exhibited the presence of acicular crystals besides the cubic ones. A punctual EDS analysis was conducted on the cubic and acicular specimens (see Supporting Information), and the results revealed that although aluminum was found in the cubic crystals, the acicular crystals presented higher aluminum amounts. Since no other crystalline phases were identified by XRD, it can be assumed that both crystals correspond to Al-rich burtite.

In general, although special care is taken during the synthesis, the nominal and real chemical compositions may differ. For that reason, the chemical composition must be determined; Table 4 presents the EDS analysis results. The presence of carbon by EDS and the symmetric and asymmetric carbonates identified in the FTIR analysis (see Supporting Information) fit with the identification of calcium carbonate (calcite) in the XRD measurements. Although a nominal Ca/Al molar ratio of 2 was fixed, the real values oscillated between 0.6 and 1.9. At the reaction conditions used in this work, it is confirmed that as the tin content augmented, aluminum precipitation is preferred over calcium, revealing that the highest tin amount that can be incorporated is 23.2%; beyond this value, aluminum-rich burtite crystallization is favored. This effect is in good agreement with the micrographs, since greater amounts of acicular particles were observed as the tin content increased.

**Table 4:** Elemental Percentages for Energy Dispersive Spectroscopy

	Element %					Molar ratio		
Sample	C	O	Ca	Sn	Al	Ca/Al	Ca/Sn	Sn/Al
Al-B0	15.0	56.9	19.7	-	8.4	1.6	-	-
Al-B1	17.1	53.3	15. 7	8.3	5.6	1.9	5.6	0.34
Al-B2	13.7	51.8	13.5	15.9	5.1	1.8	2.5	0.71
Al-B3	-	53.9	14.5	23.2	7.3	1.3	1.9	0.73
Al-B4	-	58.4	9.0	20.4	10.5	0.6	1.3	0.44

### 3.5 UV–Vis Diffuse Reflectance Spectroscopy

The UV–Vis diffuse reflectance spectra for the synthesized samples are provided in Figure 3. As can be observed, the samples exhibited several absorption edges. For instance, Al-B0 and Al-B1 showed two absorption edges at ∼225 nm and ∼310 nm, where the first one was assigned to hydrocalumite with a band gap energy of 5.01 ± 0.06 eV, while the second one was related to katoite with a band gap energy of 3.74 ± 0.02 eV.^32^ On the other hand, Al-B2, Al-B3, and Al-B4 showed three well-defined absorption edges at approximately 190, 210, and 240 nm, attributed to estimated transition energies of 5.90, 4.98 ± 0.42, and 4.36 ± 0.09 eV, respectively. These optical transitions occur within the energy range associated with burtite-based materials; however, in the multiphase Al-B2 sample, contributions from the coexisting katoite phase cannot be excluded. However, it is important to stress that Al-B3 and Al-B4 exhibited a fourth soft, constant absorption edge at 275 nm for Al-B3, and at 290 nm for Al-B4. The results are in agreement with the DRS spectra reported by Meng et al.,^9^ which also report different absorption edges for burtite. Since optical properties can be responsible for the observed photocatalytic activity, it is worth noting that all the synthesized samples exhibited multiple absorption edges; this is primarily attributed to the formation of solid solutions of burtite-katoite in Al-B1 and Al-B2. Nevertheless, the samples Al-B0, Al-B3, and Al-B4 did not exhibit the formation of a secondary phase; here, multiple absorption edges can be attributed to defect-induced electronic states within the forbidden band, including oxygen vacancies, trapped charges, and polarons.^33^

**Figure 3:**
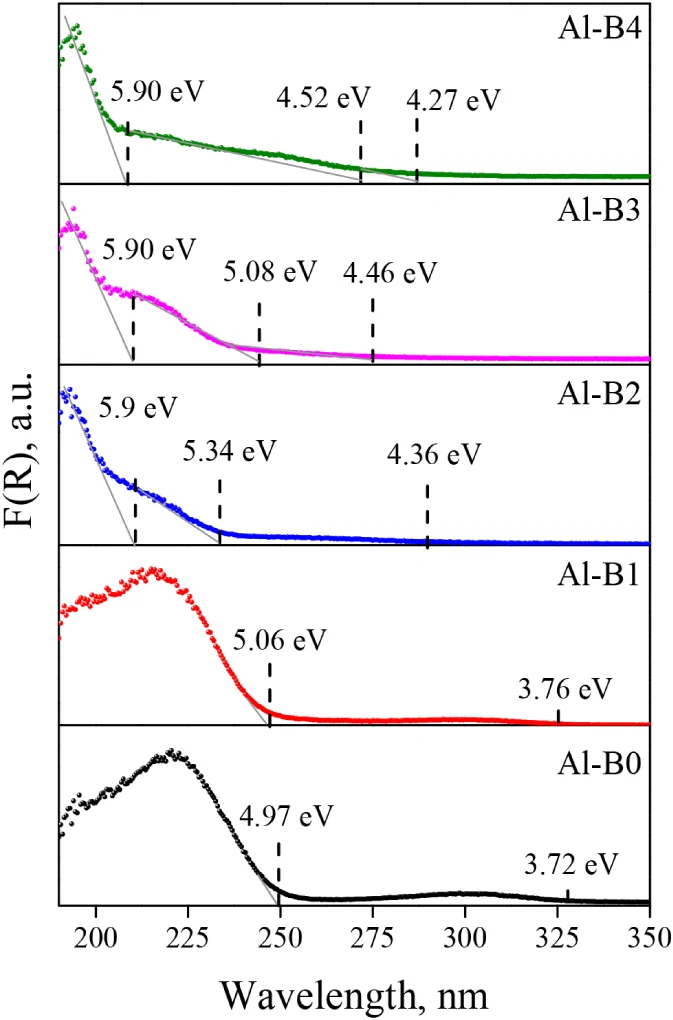
Diffuse reflectance spectroscopy study of the indicated samples.

Therefore, the additional optical transitions observed in these samples may be related to electronic states associated with structural or compositional modifications of the burtite-like lattice. Defect-related states could also contribute to these optical features; consequently, these optical features should be considered as possible contributors to materials’ photoresponse rather than direct evidence of enhanced charge-carrier separation or transfer.

### 3.6 X-ray Photoelectron Spectroscopy

The XPS spectra of the synthesized materials are shown and discussed in detail in the Supporting Information section. Figure S4 shows a comparative XPS study of the Al-B0 and Al-B2 samples, because Al-B2 will be demonstrated later to exhibit the best photoactivity. As can be seen, Gaussian deconvolution for the core levels C 1s, O 1s, Ca 2p, Al 2p, and Sn 3d was determined for each sample.

### 3.7 Photocatalytic Activity

#### 3.7.1 Photocatalysis

Initially, photolysis (UV light irradiation without a photocatalyst) and adsorption (with a photocatalyst load and UV irradiation absent) experiments were conducted. The preliminary adsorption–desorption experiment was initially conducted over 6 h using a representative catalyst to determine the equilibrium time. Since no significant changes in the UV–vis spectra were observed after approximately 1 h, this equilibration time was subsequently adopted for all photocatalytic experiments prior to UV irradiation. Further details are provided in the Supporting Information section (Figure S5A). The DiPhe spectrum showed three principal absorption bands located at 210.5, 245, and 283 nm; the first two bands are attributed to the aromatic group, and the last one is distinctive by the transition n → π* produced by the C–Cl bond.^34^ During photolysis, the absorbance values of the characteristic bands of DiPhe decreased as the UV light irradiation time increased. This effect can be attributed to the molecule degradation^35^ and to the formation of intermediates during the process, such as dichlorophenolate.^36^ Nevertheless, this process is lengthy, demonstrating that UV radiation is insufficient to achieve effective degradation.

On the other hand, the adsorption experiment for Al-B0 under dark conditions did not present evidence of adsorption (Figure 5SB). Nevertheless, over time, the intensity of the absorbance bands increased and was displaced toward longer wavelengths. This effect can be attributed to the formation of intermediates such as chlorinated *p*-benzoquinones.^37^

Photocatalytic experiments are shown in Figure 4. Prior to beginning the photocatalytic evaluation, the adsorption behavior of the photocatalyst in the dark was studied for about 1 h; for each synthesized sample, this is reported as “Ads”. Al-B0 was initially tested to determine its activity toward DiPhe photodegradation. Results indicated that although the absorbance band centered at 278 decreased consistently, the other bands showed a continuous increase over time, which is consistent with the formation of intermediates.

**Figure 4:**
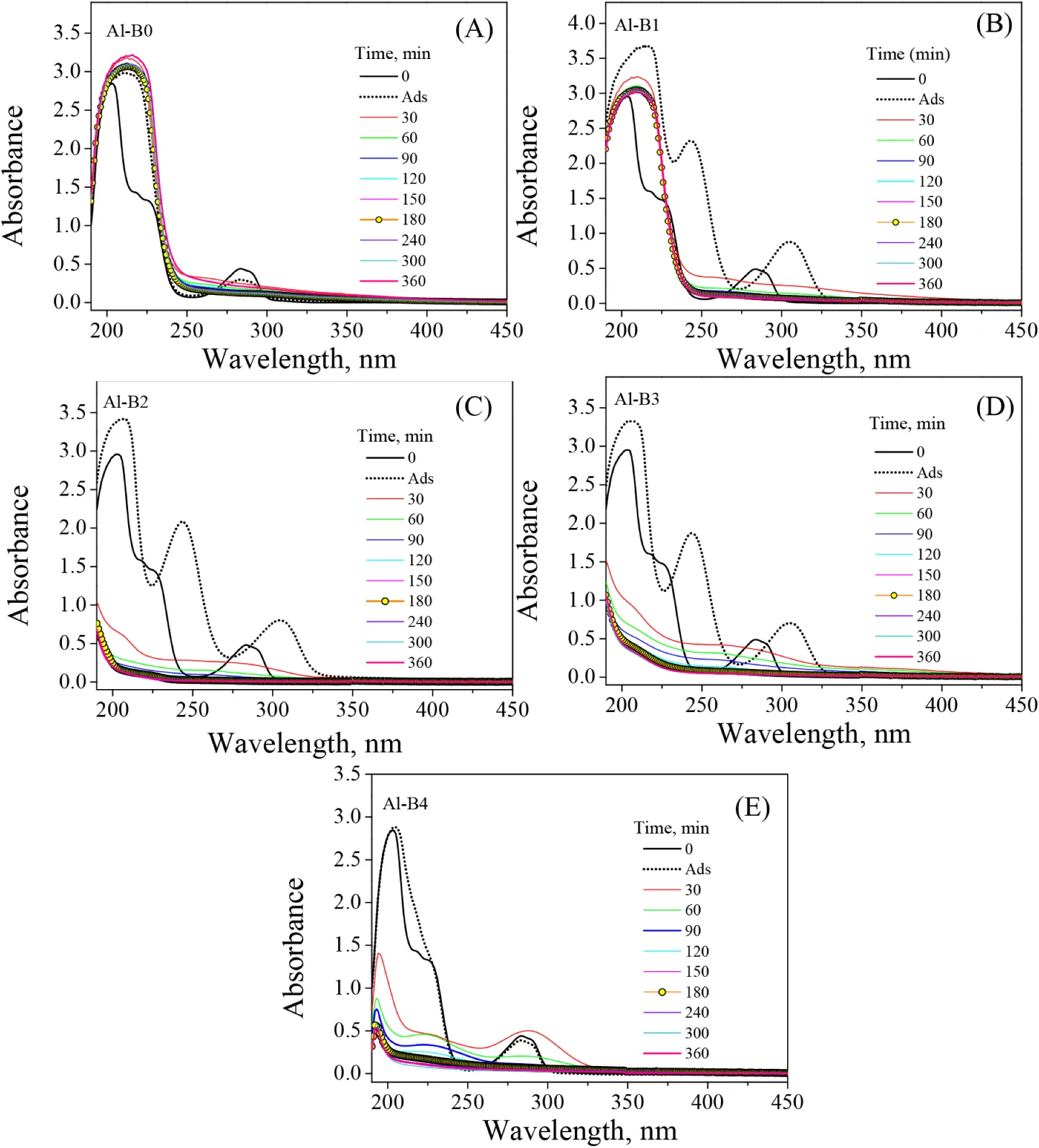
Time evolution of the absorbance spectrum of the aqueous DiPhe solution using (A) Al-B0, (B) Al-B1, (C) Al-B2, (D) Al-B3, and (E) Al-B4.

Three new absorption bands at 210, 250, and 312 nm were observed during DiPhe photocatalytic degradation. The band at 210 nm is attributed to low-molecular-weight carboxylic acids formed after ring-opening.^38^ The band at 250 nm corresponds to hydroxylated phenolic intermediates such as catechol and hydroquinone,^39^ while the band at 312 nm is assigned to quinone-type compounds (e.g., *p*-benzoquinone), which are well-known oxidation products in advanced oxidation processes.^40^

The degradation effect with Al-B2 is shown in Figure 4C; adsorption results were similar to those achieved with Al-B0. The evaluation demonstrated that the degradation effect is significant, with the three bands decreasing substantially within the first 30 min, reaching a low concentration of around 90 min. The experiment continued until a total reaction time of 360 min was reached. However, over the last 270 min, the UV–vis spectra remained almost unchanged, indicating that the percentage of degraded DiPhe in this period was minimal compared with the first 90 min.

In this sense, to highlight the differences between intermediate formation and photodegradation of the aromatic group, a 1 h lapse study was conducted; the UV–vis spectra can be consulted in SI (Figure S6). On the other side, Figure 5 shows how the concentration was modified through time using the bands at 283 and 210.5 nm. However, these UV–vis-derived profiles should be interpreted cautiously, since the recorded spectra reflect the combined absorbance contributions of DiPhe and its reaction intermediates. Consequently, the absorbance changes cannot be unequivocally assigned to the DiPhe concentration throughout the reaction. The presented UV–vis data were used primarily for comparative monitoring of the combined evolution, whereas selective identification and quantitative determination of DiPhe and individual intermediates require specific analyses, such as HPLC.^41^

**Figure 5:**
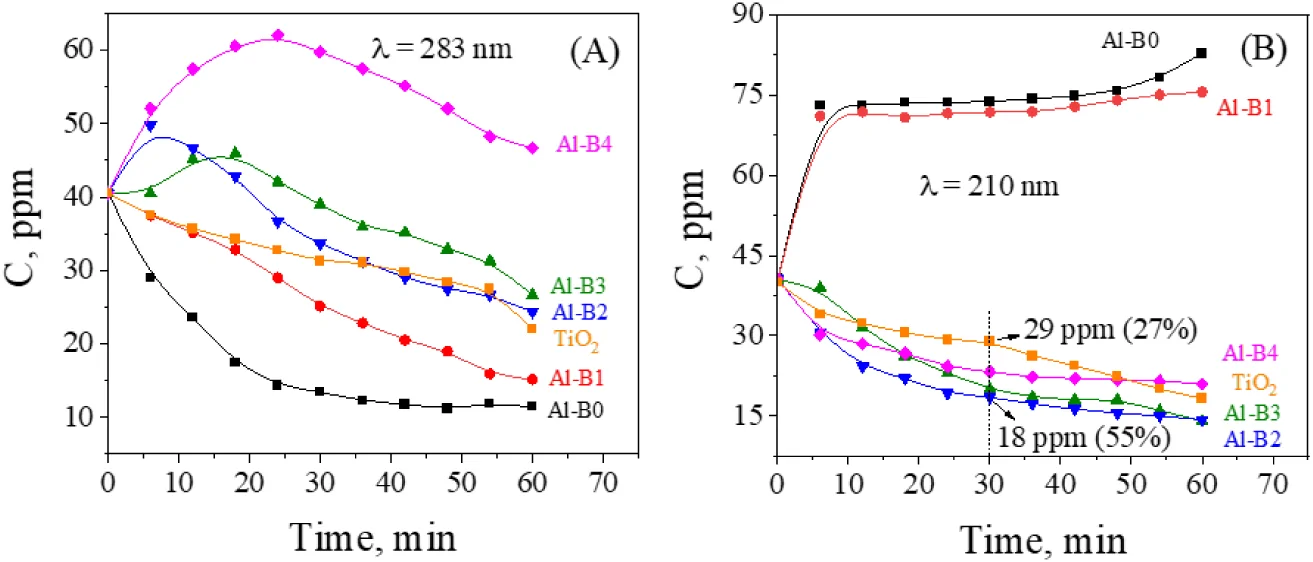
(A) Photocatalytic decomposition of DiPhe monitored at 283 nm; (B) photocatalytic decomposition of the aromatic group at 210 nm.

Thus, assigning the band at 283 nm to degradation, the results indicate that Al-B0 and Al-B1 apparently are the most active photocatalysts, achieving final DiPhe concentrations of 11 and 15 ppm after 60 min, respectively. However, reaction intermediates may also contribute to the absorbance at this wavelength; thus, these values should not be interpreted as unequivocal DiPhe concentrations. On the other hand, Al-B2, Al-B3, and Al-B4 exhibited an initial increase in absorbance at 283 nm during the first 30 min. This behavior is more appropriately attributed to spectral overlap caused by reaction intermediates rather than to an actual increase in DiPhe concentration. Then, the band’s intensity for Al-B2, Al-B3, and Al-B4 decreased continuously, reaching DiPhe concentrations of 24 and 26 ppm for Al-B2 and Al-B3, respectively, at 60 min. For Al-B4, only intermediate compounds were formed, with a final apparent DiPhe concentration of 46 ppm.

Changes in the band near 210 nm provide complementary information about the evolution of aromatic species during the reaction. In this context, although Al-B0 and Al-B1 exhibited a high photodegradation rate (determined by the UV–vis absorbance at 283 nm), the band increase at 210 nm could indicate that aromatic intermediates were not affected and that they were accumulated during irradiation, contrary to Al-B2, Al-B3, and Al-B4, which demonstrated a more pronounced decrease in this spectral region over time.

Table 5 presents the apparent degradation percentages and apparent pseudo-first-order kinetic parameters derived from the absorbance variation at 283 nm. These results were determined from Figure 5A, and the apparent pseudo-first-order rate constant (*k*_app_) was estimated from the slope of ln­(*A_t_*/*A*_0_) vs *t*, where *A_t_* and *A*_0_ correspond to the absorbance measured at 283 nm at time *t* and at the beginning of irradiation, respectively. The apparent rate constant is an indirect measure of DiPhe photodegradation. According to Table 5, Al-B1 showed the highest rate constant at 283 nm, with 208.2 × 10^–4^ min^–1^. Nevertheless, this catalyst showed a high formation of nondegraded intermediates, as observed in the degradation percentage at 210.5 nm. The second-highest apparent rate constant was exhibited by Al-B2 (116.1 × 10^–4^ min^–1^). Even though the degradation percentage at 283 nm was lower, it proved to be photoactive for aromatic group decomposition, reaching 65% at 210.5 nm. Similar behavior was observed for Al-B3, but it showed a lower degradation percentage at 283 nm (33% vs 38% compared to Al-B2).

**Table 5:** Kinetic Rate Constants for DiPhe Photodegradation and Degradation Percentages of DiPhe and Its Intermediates, Determined after 1 h of Reaction Time

Sample	*k*_app_ × 10^–4^ 283 nm	*r* ^2^	Degradation at 283 nm (%) DiPhe	Degradation at 210 nm (%) intermediates
Photolysis	88.0	0.9906	10	8
Al-B0	88.7	0.7510	72	0
Al-B1	208.2	0.9842	62	0
Al-B2	116.1	0.9728	40	65
Al-B3	101.8	0.9765	33	65
Al-B4	84.9	0.9719	0	47
TiO_2_	117.0	0.9712	45	47

It should be noted that the photocatalytic experiments were performed under monochromatic UVC irradiation (254 nm) without the use of a cutoff filter. Consequently, as shown in Figure S5, although direct DiPhe photolysis occurs under these conditions, it is demonstrated that the synthesized catalysts substantially accelerated the degradation process, reducing the time required for complete degradation, thereby confirming their photocatalytic contribution.

Even though Al-B0 showed a high degradation percentage (75%), this result was assigned to the formation of intermediates. On the other hand, the sample Al-B4 showed a gradual increase in absorbance over the first 30 min (see Figure 5A), indicating a high level of intermediates’ formation. After the first 30 min, the intermediates and aromatic groups began to degrade, reaching 47%.

Thus, the sample with the best photocatalytic behavior is Al-B2. Based on the characterization results, the high photocatalytic activity of this sample can be ascribed to a synergistic effect between katoite and burtite, since the sample presented the lowest specific surface area and similar band gap values compared to the other samples.

The photocatalytic results were compared with Degussa P-25 (TiO_2_), which is reported as a high photoactive material under UV irradiation. Figure 5A shows the concentration profile as a function of time. As can be observed, Al-B2 photodegraded the DiPhe molecule almost as effectively as TiO_2_; both materials degraded the DiPhe molecule by 40–45%.

Nevertheless, Figure 5B shows the real importance and novelty of the synthesized materials, as it demonstrates that, at 30 min, samples with higher tin content are more photoactive in breaking the aromatic ring in intermediate compounds. Al-B2, the most photoactive sample, photodegraded 56% of the intermediate compound’s molecules at 30 min, far outpacing the 25% degraded by TiO_2_.

#### 3.7.2 Mineralization Analysis by Total Organic Carbon

Total organic carbon (TOC) analysis was performed to assess the catalysts effectiveness in the mineralization of the DiPhe molecule. Figure 6 presents the mineralization percentages at 120 and 180 min. It is worth noting that Al-B2 achieved a higher mineralization value (75.3% and 76%) compared to TiO_2_ (53.8% and 56.8%) at 120 and 180 min, respectively. These results indicate that under the experimental conditions employed, Al-B2 promoted a higher degree of organic carbon mineralization than TiO_2_.

**Figure 6:**
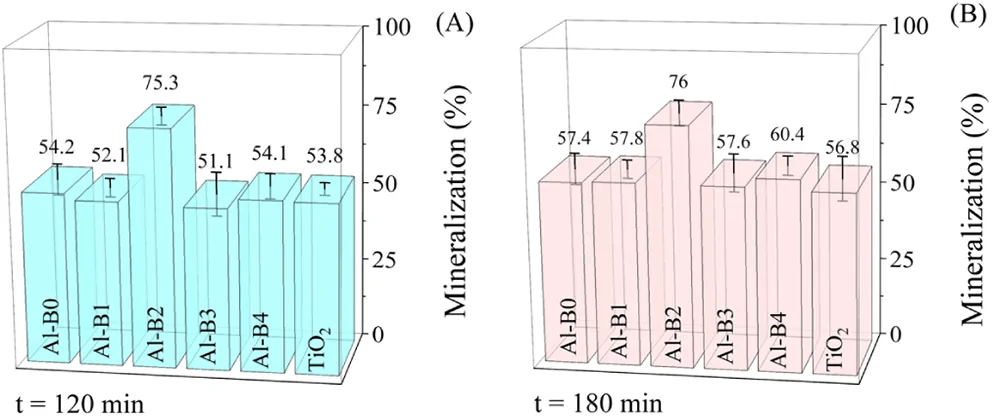
Mineralization percentages at (A) 120 min and (B) 180 min, determined by total organic carbon analysis for all the synthesized samples.

#### 3.7.3 Photocatalyst Stability

Catalyst reusability was systematically evaluated using the material that presented the best photocatalytic behavior, Al-B2 (Figure 7). The initial run (U1) and subsequent cycles or reuses (R1, R2, R3, R4, and R5) were conducted as follows: during the first run (conducted for 2 h), no aliquots were withdrawn, and at the end of the evaluation, the catalyst was removed by centrifugation in an Eppendorf tube. For the subsequent run, a new DiPhe solution (40 ppm) was added to the tube, and both were poured into the reactor to start a new evaluation.

**Figure 7:**
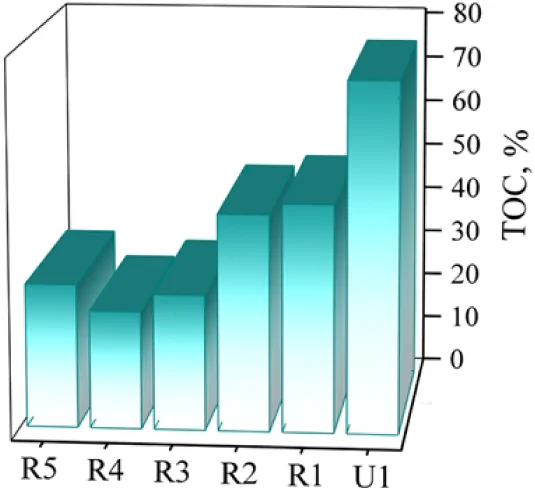
DiPhe mineralization percentages achieved in the reusability study.

From the reusability tests, it was verified that the catalyst lost its photocatalytic activity progressively from 75 to 25 TOC percentages in the first and sixth evaluations, respectively.

An important study to understand the catalyst performance after its consecutive uses (especially when deactivation is noticed) is given by its structural modification.^42^ An experiment regarding catalyst recovery after the 6th evaluation was carried out, and it was characterized by the XRD analysis. The most active material Al-B2 was selected for this study (see Figure 8). After the last evaluation, the catalyst was recovered by filtration and dried at 100 °C overnight (it was labeled as Al-B2R6). It was demonstrated that the characteristic reflections of katoite disappeared while burtite’s reflections presented a broadening effect and a shift toward higher 2-theta angles. The average crystal sizes (ACS) and the *a* cell parameter of both the pristine Al-B2 and Al-B2R were determined from the 200 plane, and the results revealed that an ACS value decreased from 59 to 34 nm while the *a* value increased from 8.089 to 8.169 Å. These results indicated that a lixiviation process was conducted throughout the evaluation cycles due to the ACS value’s decrease and the lattice expansion attributed to aluminum extraction from burtite’s lattice. The reason for this lixiviation process can be explained in terms of the DiPhe solution’s pH value. It was measured before the photocatalytic evaluation, and the results revealed that DiPhe solution exhibited acidic values (pH = 4.90). In this sense, it is reported that katoite is stable at high pH values,^23^ thus, under these acidic reaction conditions, its lixiviation can be expected.

**Figure 8:**
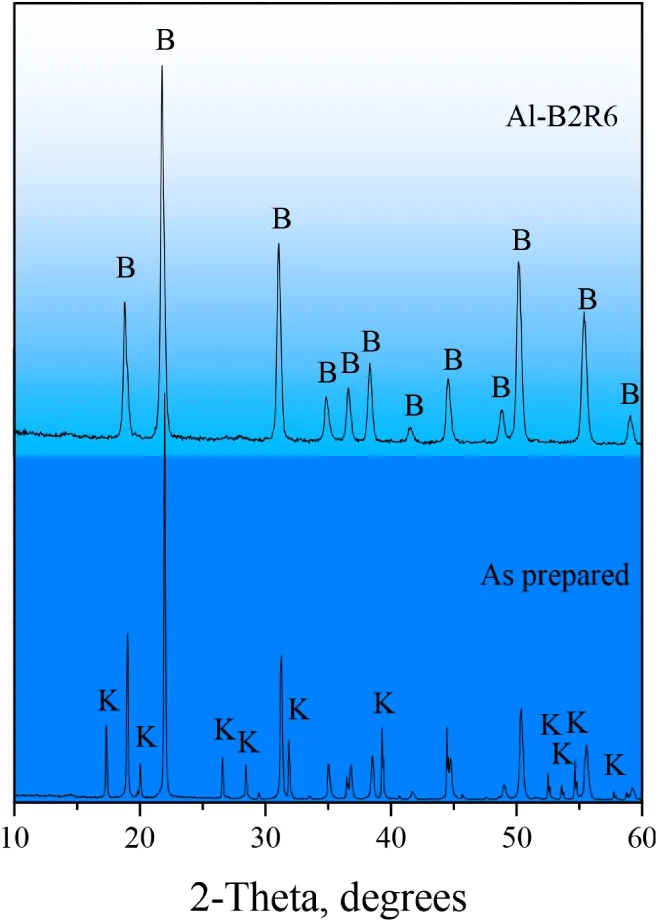
Comparison of X-ray diffraction before and after the study of photocatalyst reusability.

### 3.8 Reaction Mechanism

To explore the mechanism of DiPhe photodegradation, the formation of active hydroxyl radicals (•OH) from Al-B2 was monitored under UV irradiation by measuring the formation of terephthalic acid.

The fluorescence spectra for the terephthalic acid photolysis and the respective evaluations of Al-B2 and TiO_2_ are shown in the SI (Figure S8). The spectra showed the characteristic photoluminescent signal of hydroxyterephthalate at 425 nm.^43^ Nevertheless, the highest photoluminescence intensity was exhibited by TiO_2_, reaching a maximum of 2705, whereas Al-B2 achieved 529 and photolysis 186, respectively. Figure 9A illustrates the evolution of photoluminescence intensity in the established tests. According to Qu *et al*. and Mendoza-Damián *et al*.,^43,44^ the photoluminescence intensity is directly proportional to hydroxyl radical formation, and its formation rate can be indirectly determined by calculating the slope of the linear increase in photoluminescence.

**Figure 9:**
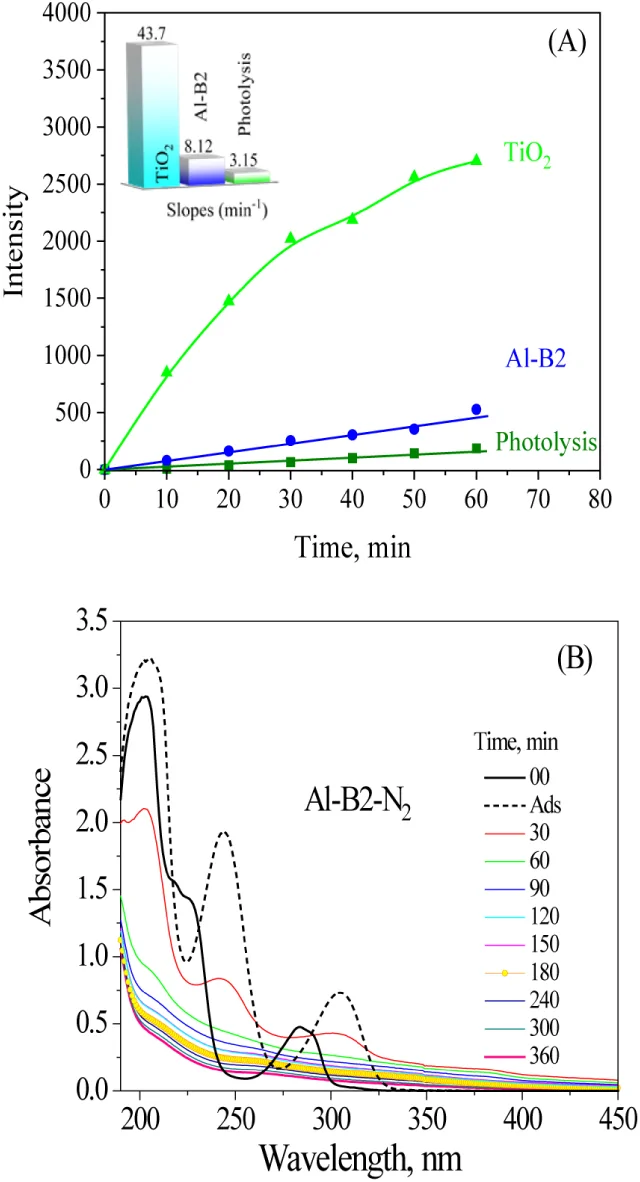
(A) Evolution in the photoluminescence intensity of terephthalic acid solution upon UV irradiation in the presence and absence of Al-B2 and TiO_2_. (B) UV–vis spectra in an oxygen-deficient aqueous medium (without air flow and using water that had previously been boiled).

The results in Figure 9A suggested that Al-B2 forms hydroxyl radicals and that the formation rate is 2.6 times higher than that of photolysis. Instead, for TiO_2_, the formation was 5.4 times higher than that in Al-B2.

On the other hand, Figure 9B shows the UV–vis spectra assessment in which the amount of oxygen in the aqueous medium was intentionally decreased by using water that had been previously boiled under N_2_ gas bubbling prior to the reaction. This test allows an indirect measurement of the superoxide radical $\left({\cdot O}_{2}^{-}\right)$ contribution to DiPhe photodegradation, where a low oxygen concentration implies a low level of superoxide radical formation. In SI section (see Figure S9), a comparison of the UV–vis spectra for DiPhe photodegradation in enriched-oxygen and low-oxygen media is provided. In general, as Table 6 shows, degradation in low-oxygen media was slower with an apparent kinetic constant of 67 × 10^–4^ min^–1^, while in an enriched-oxygen media it was of 116 × 10^–4^ min^–1^, which is 1.7 times higher.

**Table 6:** Comparison of Kinetic Parameters for DiPhe Degradation in Enriched-Oxygen and Low-Oxygen Media, Determined after 1 h of Reaction

Sample	*k* × 10^–4^ (min^–1^) 283 nm	*r* ^2^
Al-B2–O_2_	116.1	0.9728
Al-B2–N_2_	67.0	0.9973

Both results indicate that, in the presence of Al-B2, the DiPhe molecule is not preferentially degraded in one way, as is reported for TiO_2_ (where photodegradation proceeds via hydroxy radical). These results suggest that both •OH and oxygen-dependent reactive species may contribute to DiPhe degradation over Al-B2. The formation of hydroxyl radicals was lower than in TiO_2_, suggesting that superoxide radicals are responsible for photodegradation. However, when the reaction took place in a medium with low oxygen concentration, the Al-B2 material also degraded the DiPhe molecule. Therefore, it is plausible that both species are involved.

Based on the combined characterization and photocatalytic results, a plausible reaction pathway is proposed in Figure 10. As shown by XRD and SEM characterization, the most active material (Al-B2) is composed of burtite and katoite. The higher photocatalytic performance of Al-B2 compared with that of the predominantly single-phase burtite samples suggests that the coexistence of katoite and burtite contributed to its enhanced photoactivity. In this sense, UV light irradiation could promote the presence of photogenerated electrons in both materials. However, it is very probable that in katoite with a band gap of 3.0 eV, hole formation (h^+^) was superior to that in burtite.

**Figure 10:**
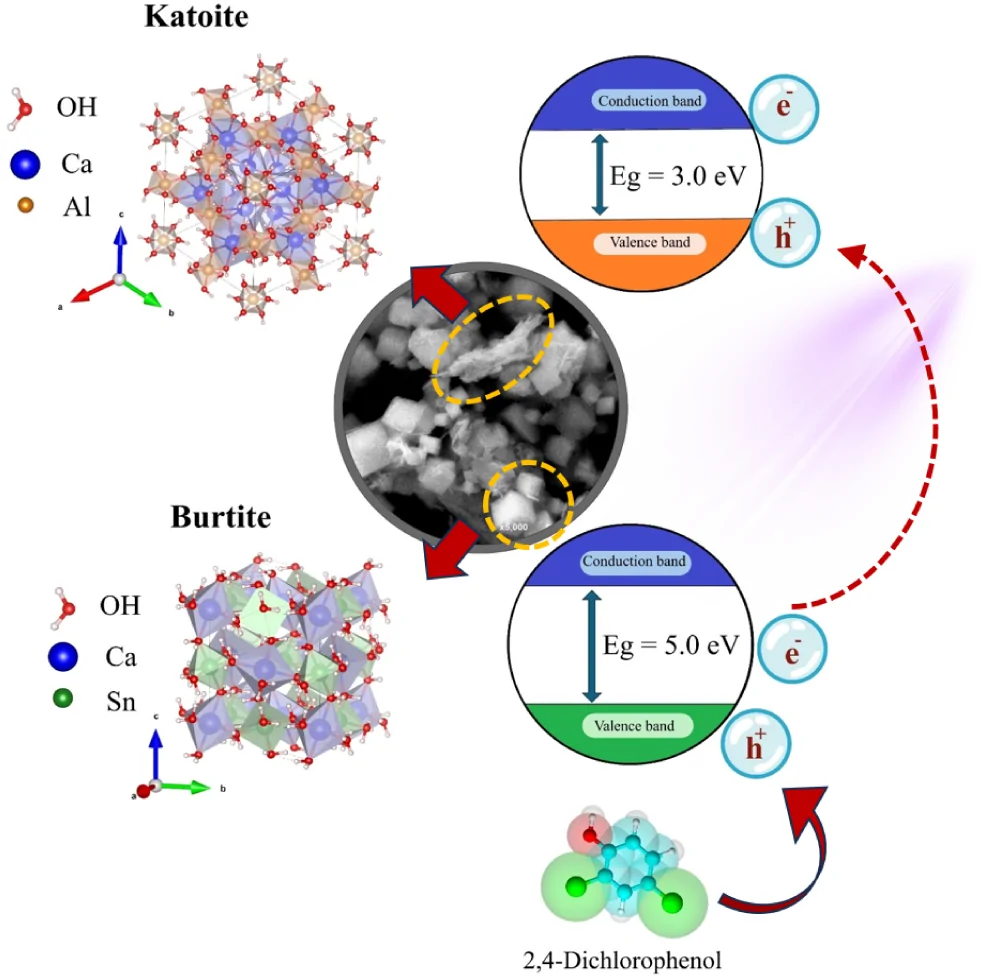
Schematic representation of the possible pathway for 2,4-dichlorophenol photodegradation.

According to the literature, the irradiation energy must be equal to or preferentially higher than the band gap energy in order to favor the photoexcitation of the electrons (e^–^) from the valence band (VB) toward the conduction band (CB), giving rise to hole formation (h^+^).^45^ In this case, the lamp emitting at 254 nm provides photons with an energy of 4.9 eV. Hence, the availability of holes in burtite may be limited since the photogenerated electrons would not have sufficient energy to reach the conduction band. Therefore, rapid recombination of the electron may be favored, returning it to its basal state or creating a negative charge density in burtite. Thus, excitation of the higher-energy transitions associated with burtite may be less favorable under these irradiation conditions. Nevertheless, the DRS results revealed multiple optical transitions in the burtite-containing samples, including features near the energy supplied by the irradiation source. Therefore, burtite’s contribution to the overall photoresponse cannot be excluded.

On the other hand, the experimental evidences indicate that the coexistence of katoite and burtite could facilitate the transfer of photogenerated electrons. The lower band gap of katoite (3.0 eV) would allow the electron–hole formation (e^–^/h^+^); therefore, holes, considered as charge vacancies, could be compensated by electron transfer from burtite.

Consequently, hole formation in burtite is indirect, as the holes do not originate from the photoexcitation of valence-band electrons. Instead, they arise from charge compensation between katoite and burtite.

Therefore, band gap engineering in both materials may enable an adequate pathway for electron transfer, hole formation, and charge separation with electrons accumulating in katoite and holes in burtite, allowing adequate charge separation, thereby minimizing electron–hole pair recombination and giving rise to the formation of highly reactive species such as •OH, h^+^, •OOH, and •O^–2^, which are directly responsible for organic molecule degradation and mineralization.^20^

## 4 Conclusions

In this work, a novel series of aluminum-containing burtite-like solid solutions was successfully synthesized via coprecipitation followed by hydrothermal treatment. To the best of our knowledge, this type of aluminum-doped burtite system has not been previously reported. By varying the tin content (5.3–27.8 mol %), the phase composition could be systematically modified, thereby stabilizing the burtite structure and eliminating secondary phases. These changes directly affected the electronic structure and band-gap energies and enhanced charge transfer.

As a result, the photocatalytic performance was significantly improved. The most photoactive material, Al-B2, achieved 56% degradation of DiPhe and its intermediates within 30 min under UV irradiation, clearly outperforming TiO_2_, which reached only 25% under the same conditions. This enhancement is attributed to the synergistic effects of phase composition, optical properties, and increased oxygen vacancy concentration. Notably, the catalyst lost its activity progressively throughout 6 consecutive evaluations (from 70 to 25 TOC %), which was attributed to its lixiviation due to the reaction medium’s acidic conditions.

Overall, these results demonstrate that aluminum-containing burtite-like solid solutions are a previously unexplored and promising class of photocatalysts for the removal of chlorinated organic pollutants from water.

## Supplementary Material



## Data Availability

The data used are available throughout the manuscript.

## Notes

Additional experimental details for material synthesis; reactant amounts and synthesis procedure; EDS, FTIR, and XPS characterization; UV irradiation, adsorption, and photocatalytic control experiments; UV–vis spectra of 2,4-dichlorophenol photodegradation and indirect determination of hydroxyl and superoxide radical formation (PDF)
