# Supplementary material for: 2,4-Dichlorophenol Photodegradation by Novel Burtite-like Solid Solutions: Effect of Sn4+ and Al3+ Content on the Physicochemical Properties and Photocatalytic Performance
Source: ACS Omega. 2026 Oct 1;11(40):60968–79. doi: 10.1021/acsomega.6c04908 (PMC13670345; doi:10.1021/acsomega.6c04908)
Supplement: Supplementary file 1 [file ao6c04908_si_001.pdf]

Supporting Information

2,4-dichlorophenol photodegradation by novel Burtite-like solid solutions: Effect of Sn^{4+} and Al^{3+} content on the physicochemical properties and photocatalytic performance

Federico Manuel Reyes-Cruz[(a)], Manuel Sánchez-Cantú[(a)]*, Clara Tzompantzi-Flores[(a)]*, Irving I. Ruiz-López[(a)], Angela-Gabriela Romero-Villegas[(b)], Francisco Tzompantzi[(b)]

[(a)] *Facultad de Ingeniería Química, Benemérita Universidad Autónoma de Puebla, Facultad de Ingeniería Química, Avenida San Claudio y 18 Sur, C.P. 72570 Puebla, Puebla, México*

[(b)] *Departamento de Química, Área de Catálisis, Universidad Autónoma Metropolitana Iztapalapa, Av. San Rafael Atlixco No. 189, Iztapalapa, 09340 México City, México*

* *To whom correspondence should be addressed:*

+ (52 55) 2295500 Ext. 7265; e-mail: manuel.sanchez@correo.buap.mx,

+ (5255) 5804 4669; e-mail: ctzompantzif@ipn.mx

Section S1. Materials' synthesis

a) Hydrocalumite

Hydrocalumite (Al-B0) was synthesized by the co-precipitation method using the reactant amounts listed in Table S1. Briefly, two solutions were prepared by separate. Solution A was prepared by dissolving $Ca(NO_3)\cdot 4H_2O$ (23.5g), $Al(NO_3)\cdot 9H_2O$ (18.9 g) salts and NH_4NO_3 (3.1g) in bi-distilled water at room temperature while maintaining a Ca^{2+}/Al^{3+} molar ratio = 2. After complete dissolution of the salts, 2 mL of HNO_3 were added and the solution was stirred for 30 min to ensure complete homogenization. By separate, solution B was prepared by dissolving KOH (19.5 g) in bi-distilled water. Solution B was then added dropwise to Solution A under continuous stirring until the pH reached 12. Afterwards, the mixture was transferred to a Parr reactor and heated to 150 °C (heat ramp 20°C min^{-1}) under continuous stirring (350 rpm) for 5h. The resulting material was filtered and washed with bi-distilled hot water and dried for 24 hours at 100 °C.

b) Burtite-like compounds

Burtite-like compounds were prepared according to the co-precipitation procedure described in Section S1a for hydrocalumite, incorporating different amounts of tin into the precursor solution. The tin content was varied from 5.3- 27.8 mol% respective to the metallic cations content. The reactant quantities used for each composition are summarized in Table S1, and the resulting materials were labelled Al-B0, Al-B1, Al-B2, Al-B3, and Al-B4, corresponding to tin contents of 0, 5.3, 10.3, 19.5, and 27.8 mol%, respectively.

A schematic flowchart summarizing the synthesis procedure for hydrocalumite and burtite-like compounds is presented in Figure S1.As a representative example, the synthesis of the material containing 5.3 mol% Sn (Al-B1) is described below.

Solution A was prepared by dissolving $Ca(NO_3)_2{\cdot}4H_2O$ (23.0 g), $Al(NO_3)_3{\cdot}9H_2O$ (18.5 g), $SnCl_4{\cdot}5H_2O$ (2.9 g), and NH_4NO_3 (3.0 g) in bi-distilled water. Subsequently, 2 mL of HNO_3 were added, and the mixture was stirred for 30 min. Separately, Solution B was prepared by dissolving KOH (19.1 g) in bi-distilled water. Solution B was then added dropwise to Solution A until the pH reached 12. The resulting suspension was hydrothermally treated at 150 °C (heating rate of 20 °C min^{-1}) under continuous stirring (350 rpm) for 5 h. The resulting material was filtered and washed with hot bi-distilled water and dried at 100 °C for 24 hours.

Table S1

Reactants amounts used for each material's synthesis.

**Sample**	$Ca(NO_3)_2$*4(H_2O), g	$Al(NO_3)_3$*9(H_2O), g	$Sn(Cl)_4$*5(H_2O), g	NH_4NO_3, g	KOH, g	HNO_3, ml
**Al-B0**	23.5	18.9	0.0	3.1	19.5	2
**Al-B1**	23.0	18.5	2.9	3.0	19.1	2
**Al-B2**	22.5	18.1	5.8	3.0	18.7	2
**Al-B3**	21.4	17.2	11.6	2.8	17.8	2
**Al-B4**	20.4	16.4	17.6	2.7	16.9	2

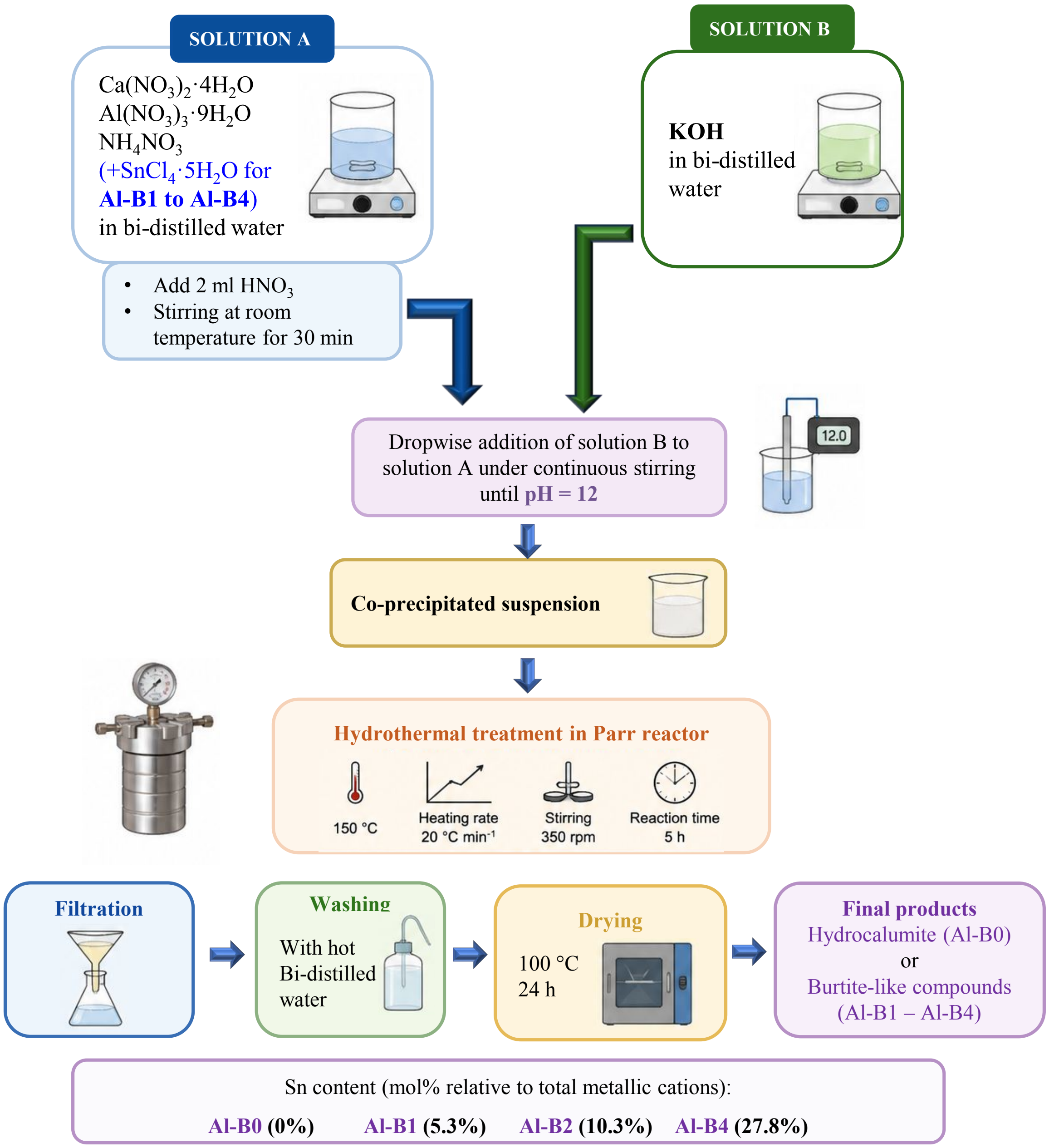

Fig. S1. Schematic representation of the synthesis procedure for hydrocalumite and burtite-like compounds.

Section S2. Morphological and elemental analysis

Complementary figures to Section 3.4 in the main document.

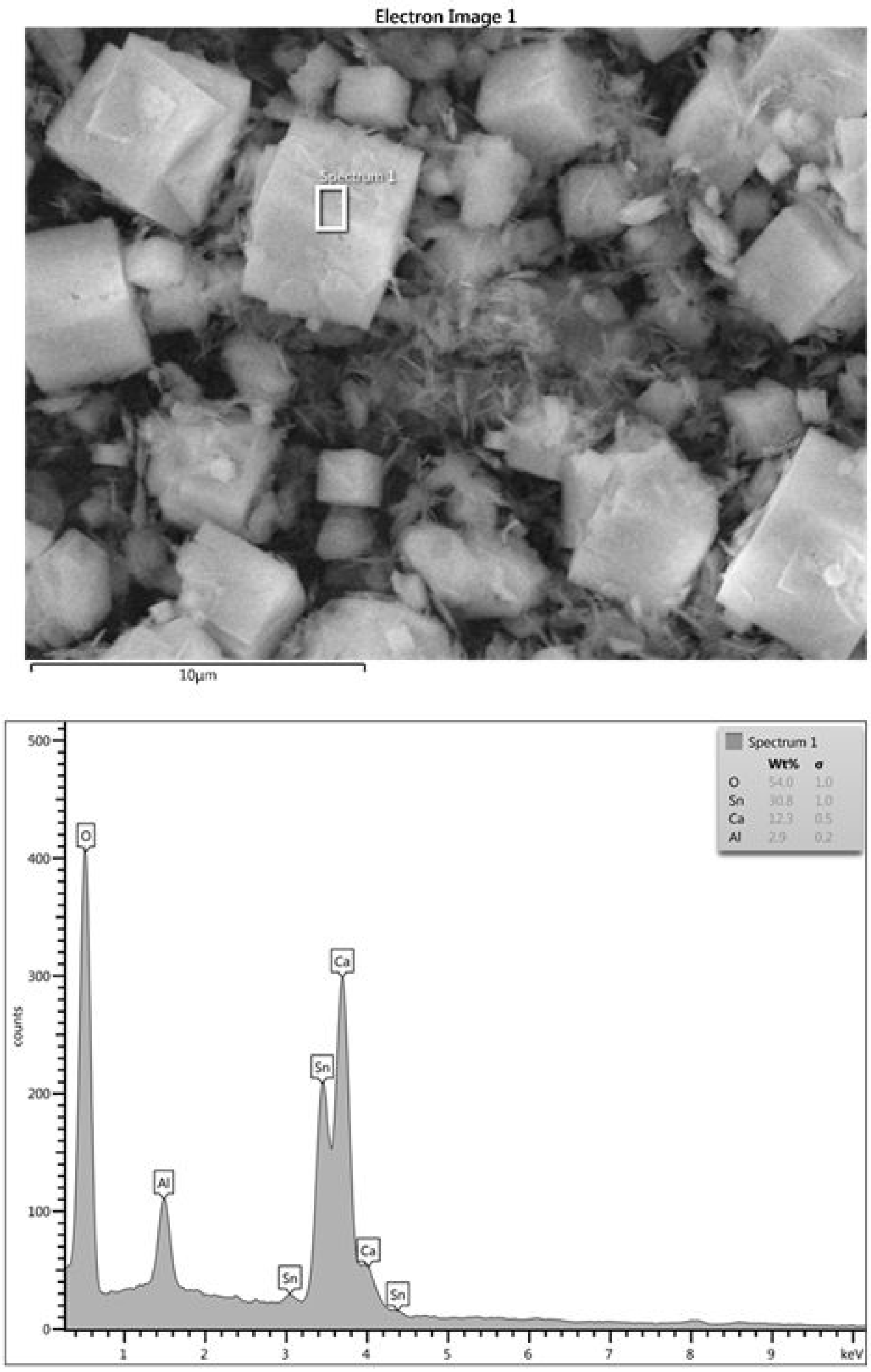

Fig. S2. EDS analysis carried out on a cubic crystal.

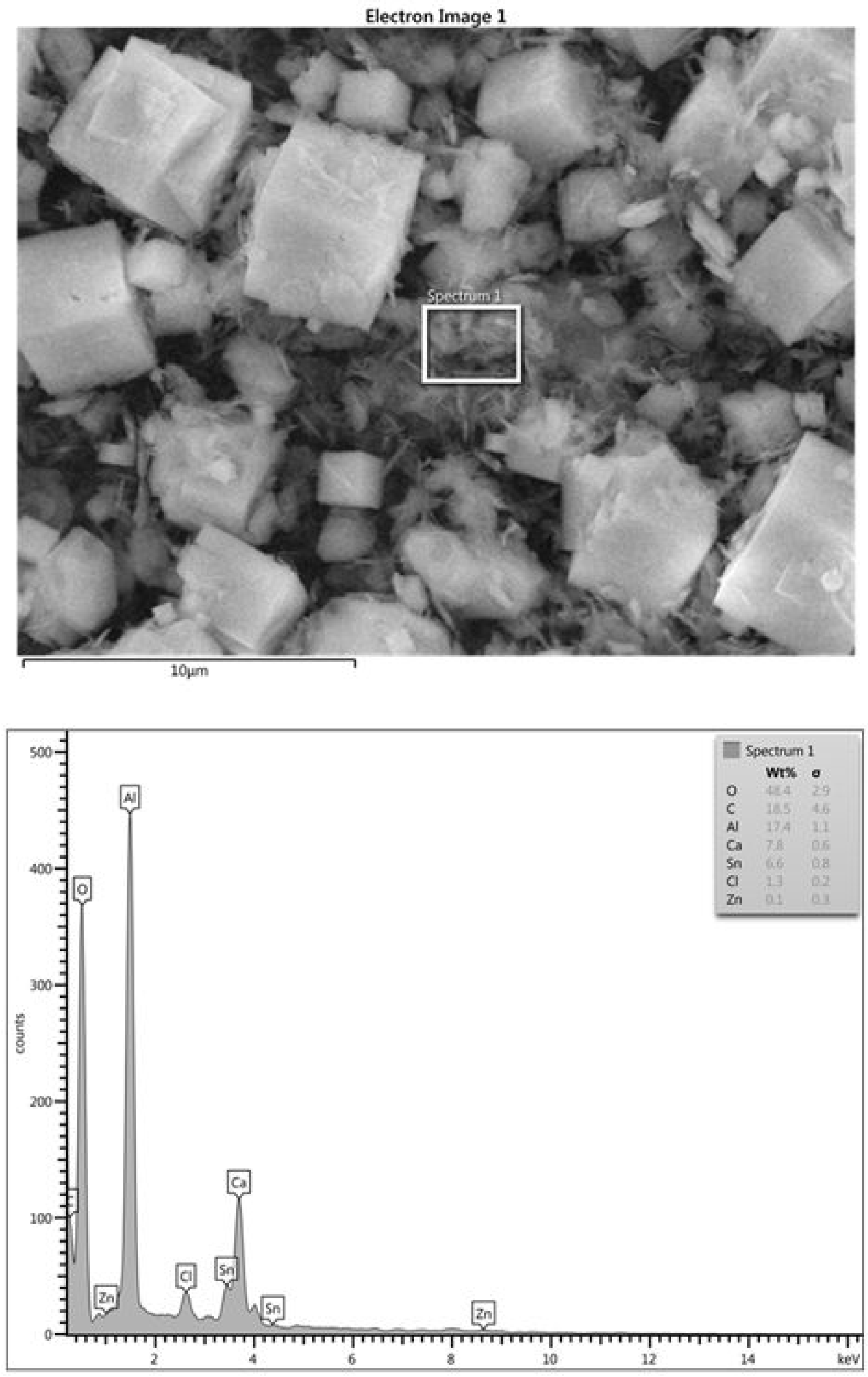

Fig. S3. EDS analysis carried out on acicular-rich crystals zone.

Section S3. Fourier Transform Infrared Spectroscopy

Figure S4 exhibits the infrared spectra of the synthesized materials. The samples showed vibrations of the free O-H stretching frequency at ~ 3600 cm^{-1} and O-H stretching at 3200-3300 cm^{-1}, characteristics of hydroxyl ions in the materials' lattice.[1]

The bands at 1373 and 1065 cm^{-1} indicate the carbonate vibration modes, namely the asymmetric stretching (v_3) and the symmetric stretching (v_1), respectively.[2] On the other hand, the vibrations at 772 and 586 cm^{-1} are attributed to Al-O.[3,4]

As can be observed in Figure S4, a clear modification is evident in the IR spectra for the high-tin-content samples. The sample Al-B0 showed characteristic vibrations of hydrocalumite at 3600 cm^{-1} of free O-H stretching, 1373 cm^{-1} of CO_3^{2-} in v_3 mode, 772 cm^{-1} of Al-O stretching vibrations, and 586 cm^{-1}, attributed to M-O-M (Ca–O–Al/Ca–O–Ca/Al–O–Al) lattice stretching vibrations.[4] The vibration at 1400 cm^{-1}, visible only for Al-B1, was indexed to the presence of carbonate in the v_3 mode from katoite,[3] which is in correspondence with the XRD study, where it was demonstrated that the sample Al-B1 is constituted by hydrocalumite and katoite.

For samples with higher tin content (Al-B2, Al-B3, and Al-B4), spectral modifications were observed. Compared to Al-B0 and Al-B1, the vibrations that were no longer observed were at 3600 cm^{-1} (free O-H), 1372 cm^{-1} (CO_3^{2-}, v_3), and 772 cm^{-1} (Al-O). At 586 cm^{-1}, corresponding to the M-O-M bond of Ca and Al, the transmittance decreased with increasing tin content in the samples. On the other side, additional vibrations appeared at 3200-3300 cm^{-1} (O-H ions in the lattice), while the transmittance of the vibration at 1065 cm^{-1} (CO_3^{2-}, v_1) apparently was slightly shifted to higher wavenumbers and increased as the tin content increased. Nevertheless, this last vibration is not indexed to carbonate species in

Al-B2, Al-B3, and Al-B4; instead, it is related to Sn-O and Sn-OH vibrations. This agrees with Gubuish et al.,[5] who reported that the characteristic vibrations for burtite are visible at 3304 cm^{-1} (O-H ions in the lattice), 1574 and 1391 cm^{-1} (carbonyl group), 1097 cm^{-1} (Sn-O and Sn-OH), and 690 and 505 cm^{-1} (M-O-M bond).

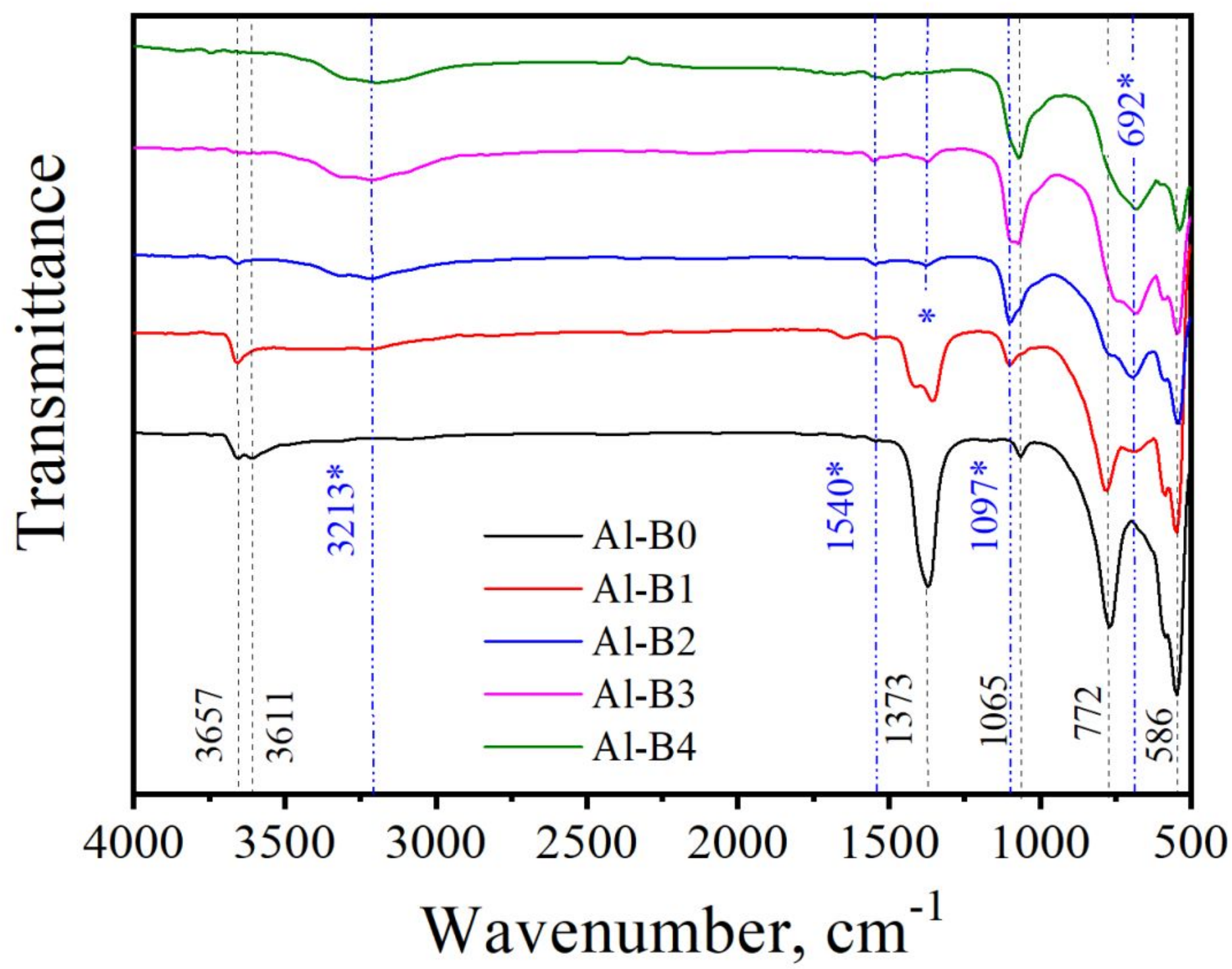

Fig. S4. IR spectra of the synthesized materials

Section S4. X- Ray photoelectron Spectroscopy

XPS survey spectra for the most photoactive sample, Al-B2, and the bare sample Al-B0 are shown in Figure S5. The samples exhibited peaks at characteristic binding energies corresponding to the core levels Ca 2s, Ca 2p, Ca 3p, Al 2s, Al 3p, O 1s, and C 1s. On the other hand, the tin-containing samples also exhibited the characteristic binding energies of the Sn 3d core level.

To study the chemical modifications, a deconvolution analysis was performed on the representative core levels C 1s, O 1s, Ca 2p, Al 2p, and Sn 3d of Al-B0 and Al-B2 (see Figure S5). These samples were selected to stress the differences between the sample without tin and the most active material. Figure S5A shows the C 1s core level for both samples, the sample Al-B0 exhibited two peaks at 284.6 and 289.4 eV assigned to adventitious carbon, AC, and hydrocarbon species, COOH, respectively. The deconvolution of Al-B2 sample also showed adventitious carbon and carbonate species C-O and C=O at 286.7 eV, as well as a satellite π-π* at 291.0 eV.[6]

Figure S5B presents the deconvolution for the core level O 1s, where both samples Al-B0 and Al-B2 exhibited three peaks: i) between 528.6 and 529.6 eV corresponding to metal oxides, ii) between 530.2 and 531.1 eV indexed to carbonate and hydroxide species,[7] and iii) between 531.5 and 532.4 related to oxygen vacancies.[8] From the analysis, it was observed that the incorporation of tin resulted in lower formation of hydroxide and carbonate species, while the presence of metal oxide and oxygen vacancy species was enhanced. The increase in oxygen vacancies is crucial because they serve as sites where the pollutant molecule can be adsorbed and then oxidized.

The core-level Ca 2p deconvolution (see Figure S5C) demonstrated that both samples showed doublets being the higher binding energy peak (350.8 eV and 351.2) attributed to the Ca 2p1/2 orbital, while the lower binding energy peak (347.0 eV and 347.7 eV) was assigned to the Ca 2p3/2 orbital; both matched the Ca-O bond [8]. Contrary to Guo et Al. reports,[9] the doublet $Ca^{(\delta-)}$ at lower binding energies (345.3 eV and 350.9 eV) can be attributed to an electron cloud density increment of Ca^{2+} by the presence of tin; a high electron abundance leads to the transfer of Ca 2p in the sample to lower binding energies. The shift observed from 347.0 eV and 350.8 eV in Al-B0 to 347.7 eV and 351.2 eV in Al-B2 was attributed to oxygen vacancies that originated an excited state Ca^{2+}.[8]

On the other hand, the deconvolution analysis for the core-level Al 2p (see Figure S5D) indicates that aluminum is present in the samples in different chemical states. Around a binding energy of 72.2 and 72.6 eV, the presence of metallic aluminum (Al^0) was evidenced in Al-B0 and Al-B2,[10] while the binding energy at 75.0 eV was related to $Al(OH)_3$.[11] Also, the binding energies around 73.5 and 73.9 eV were attributed to partially hydroxylated species $Al(OH)_x$, probably originating from superficial suboxide species, AlyOx, which can adsorb water from the environment.[12]

Finally, the deconvolution analysis of the core-level Sn 3d is exhibited in Figure S5E. The presence of three doublets was observed, with the highest-energy doublet corresponding to 3d 3/2 and the lowest-energy doublet to 3d 5/2. The doublets were assigned to metallic tin Sn^0 (484.8 and 493.2 eV), Sn^{2+} (486.2 and 494.7 eV) and Sn^{4+} (487.3 and 495.9 eV), respectively.[12]

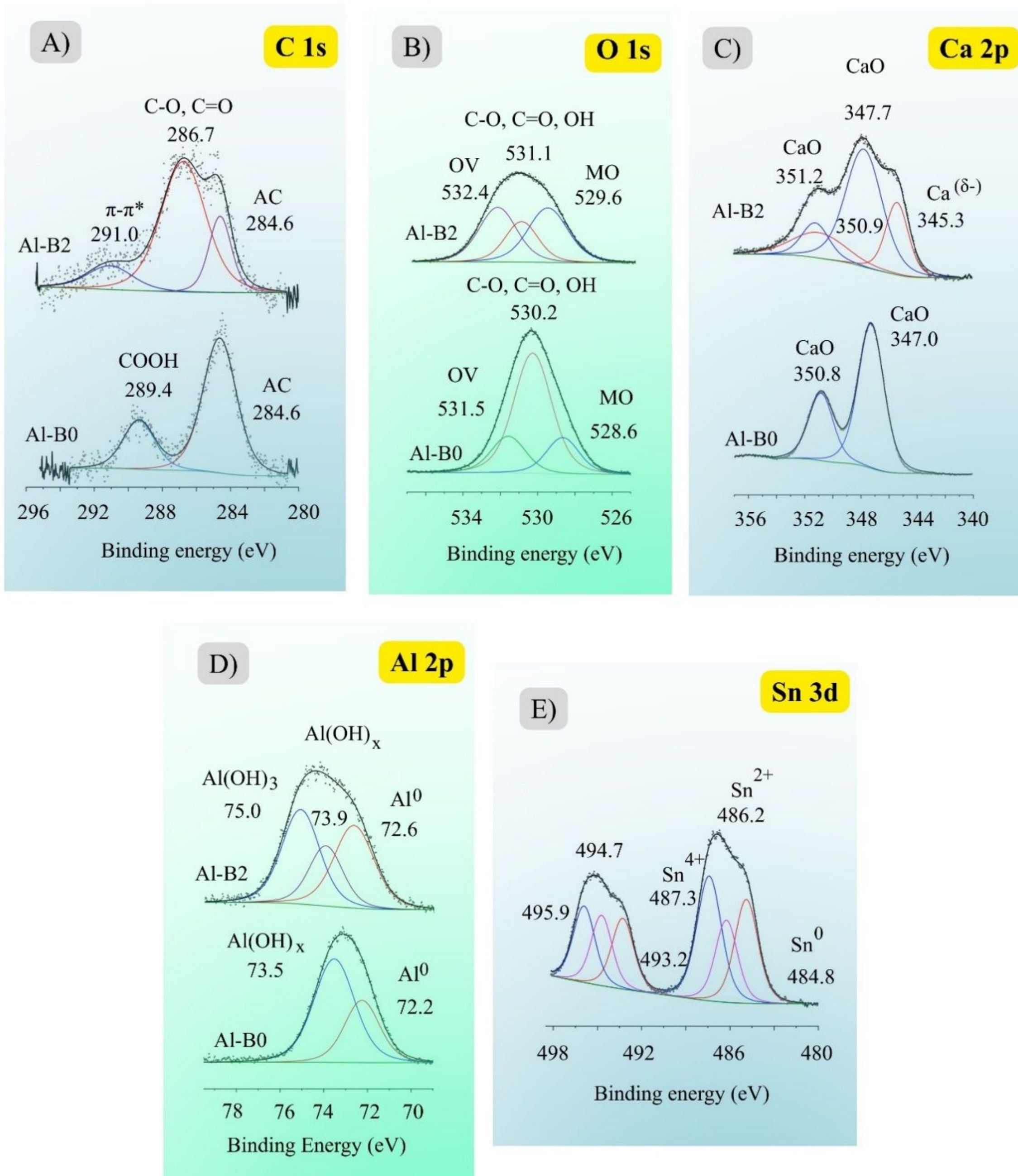

Fig. S5. XPS study for the samples Al-B0 and Al-B2; deconvolution for the core levels a) C 1s, b) O 1s, c) Ca 2p, d) Al 2p, and e) Sn 3d.

Section S5. UV light irradiation

Supplementary figure corresponding to section 3.7.1 in the main document.

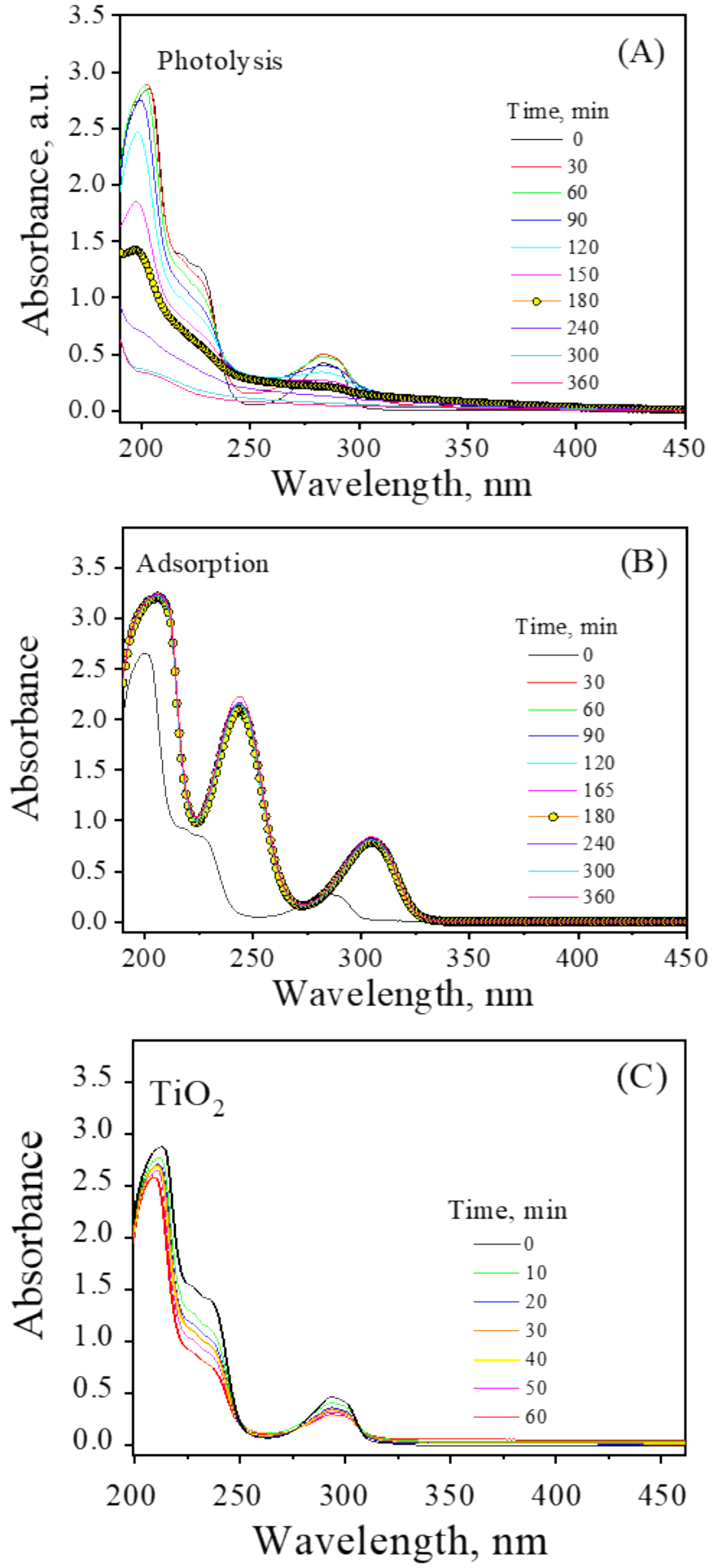

Fig. S6. (A) UV irradiation of aqueous solution without photocatalyst (photolysis), (B) adsorption test for the sample Al-B0 (without UV irradiation), and (C) DiPhe photodegradation with Degussa P25 (TiO_2).

Section S6. Photodegradation experiments to determine the kinetic rate constant

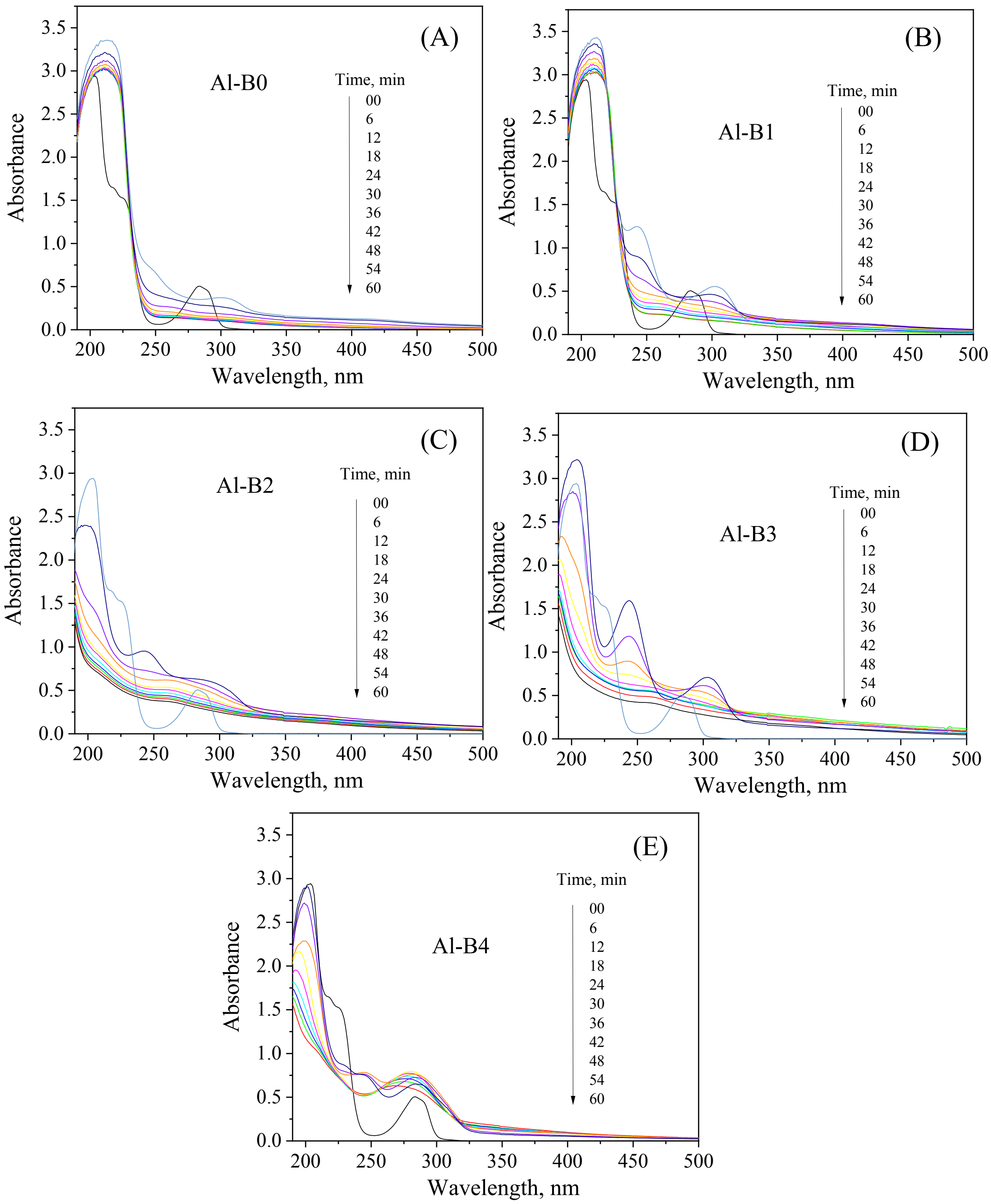

Fig. S7. UV-Vis spectra for the photodegradation of DiPhe for all the synthesized samples, after 1 h of reaction time.

Section S7. Indirect determination of •OH formation

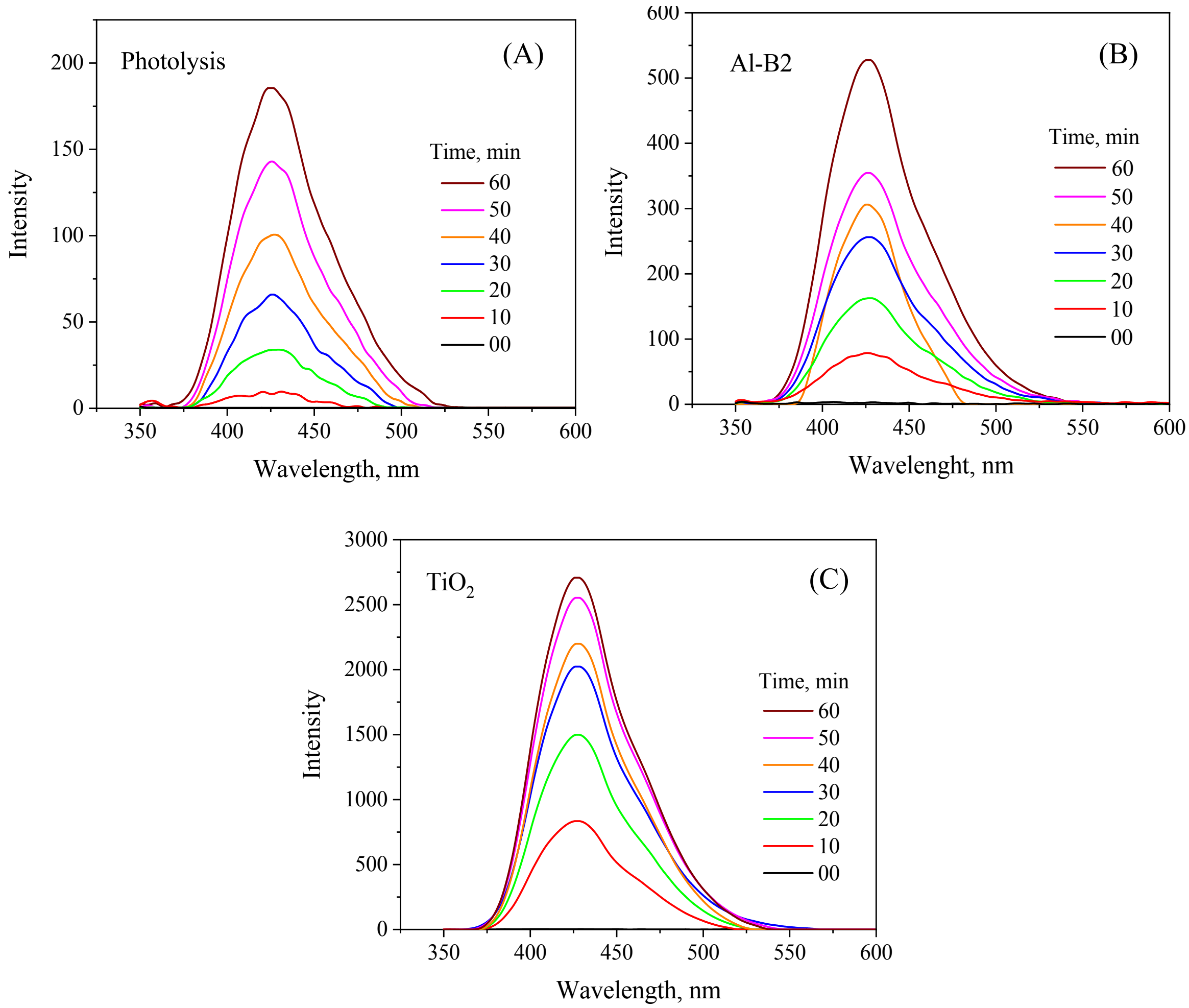

Fig. S8. Photoluminescence spectra for hydroxyl radical identification through the UV irradiation of terephthalic acid.

Section S8. Indirect determination of $\bullet O_2^-$ formation

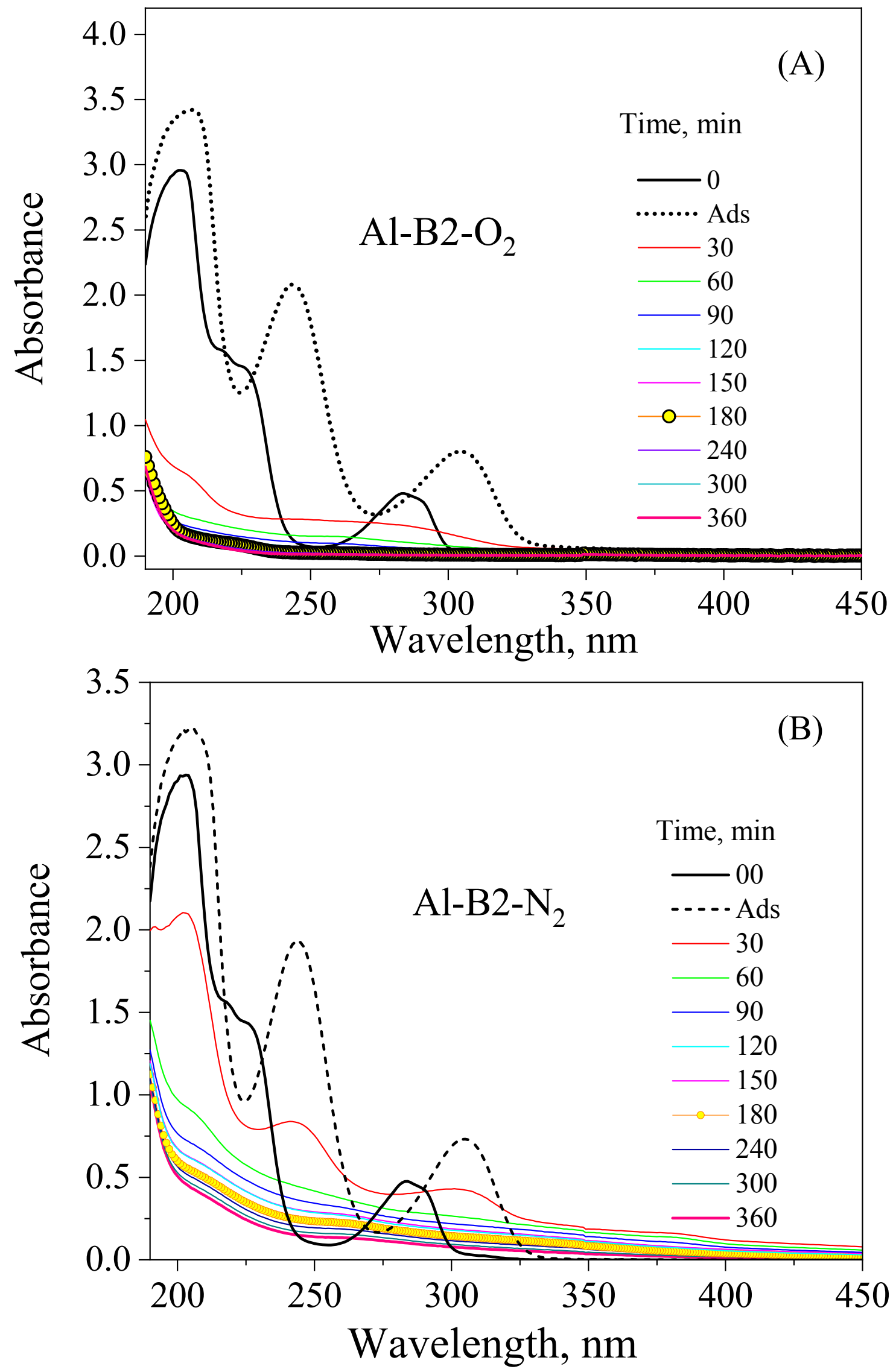

Fig. S9. UV-Vis spectra of DiPhe photodegradation in enriched-oxygen and low-oxygen-concentration media.

References

(1) Jena, H.; Kutty, K. V. G.; Kutty, T. R. N. Ionic Transport and Structural Investigations on $MSn(OH)_6$ (M = Ba, Ca, Mg, Co, Zn, Fe, Mn) Hydroxide Perovskites Synthesized by Wet Sonochemical Methods. *Mater. Chem. Phys.* **2004**, *88* (1), 167–179.

(2) Britvin, S. N.; Murashko, M. N.; Vlasenko, N. S.; Vereshchagin, O. S.; Bocharov, V. N.; Vapnik, Y. Mampsisite, $Ca_4Al_2(OH)_{12}(CO_3)\cdot 5H_2O$, a New Mineral and a New Polymorph of the Cementitious AFm Monocarboaluminate. *American Mineralogist.* **2025**, 1, 1-40.

(3) Sha, L.; Zou, Z.; Qu, J.; Li, X.; Huang, Y.; Wu, C.; Xu, Z. As(III) Removal from Aqueous Solution by Katoite ($Ca_3Al_2(OH)_{12}$). *Chemosphere.* **2020**, *260*, 127555.

(4) Prado, R. G.; Corrêa, A. G. S.; Rodrigues, J. F.; Scaldaferri, C. A.; de Sousa, F. P.; Pasa, V. M. D.; Constantino, V. R. L.; Pinto, F. G.; Tronto, J. Catalysts Derived from Thermal Treatments of Hydrocalumite-Type Compounds: Synthesis and Application in Transesterification Reactions. *J. Braz. Chem. Soc.* **2023**, *34* (1), 2–12.

(5) Ghubish, Z.; Saif, M.; Hafez, H.; Mahmoud, H.; Kamal, R.; El-Kemary, M. Novel Red Photoluminescence Sensor Based on Europium Ion Doped Calcium Hydroxy Stannate $CaSn(OH)_6:Eu^{+3}$ for Latent Fingerprint Detection. *J. Mol. Struct.* **2020**, *1207*.

(6) Chen, X.; Wang, X.; Fang, D. A Review on C1s XPS-Spectra for Some Kinds of Carbon Materials. *Fuller. Nanotube. Car. N.* **2020**, 28(12), 1048–1058.

(7) Abreu-Sepulveda, M. A.; Dhital, C.; Huq, A.; Li, L.; Bridges, C. A.; Paranthaman, M. P.; Narayanan, S. R.; Quesnel, D. J.; Tryk, D. A.; Manivannan, A. The Influence of Fe Substitution in Lanthanum Calcium Cobalt Oxide on the Oxygen Evolution Reaction in Alkaline Media. *J. Electrochem. Soc.* **2016**, *163* (9), F1124.

(8) Yuyuan, Z.; Zhiwei, G.; Haocheng, S.; Liang, W.; Shuang, Z.; Xipeng, L.; Qicheng, C.; Haisheng, C. The Role of Oxygen Vacancy in $CaO-Ca_{12}Al_{14}O_{33}$ Materials Derived from Hydrocalumite for Enhanced CO_2 Capture Cyclic Performance. *Chem. Eng. J.* **2024**, *481*, 147955.

(9) Guo, H.; Kou, X.; Zhao, Y.; Wang, S.; Sun, Q.; Ma, X. Effect of Synergistic Interaction between Ce and Mn on the CO2 Capture of Calcium-Based Sorbent: Textural Properties, Electron Donation, and Oxygen Vacancy. *Chem. Eng. J.* **2018**, *334*, 237–246.

(10) Yin, Z.; Mei, S.; Gu, P.; Wang, H. Q.; Song, W. Efficient Organic Solar Cells with Superior Stability Based on PM6:BTP-EC9 Blend and AZO/Al Cathode. *iScience.* **2021**, *24* (9), 103027.

(11) Barrera, A.; Tzompantzi, F.; Campa-Molina, J.; Casillas, J. E.; Pérez-Hernández, R.; Ulloa-Godinez, S.; Velásquez, C.; Arenas-Alatorre, J. Photocatalytic Activity of Ag/Al_2O_3-Gd_2O_3 Photocatalysts Prepared by the Sol-Gel Method in the Degradation of 4-Chlorophenol. *RSC Adv.* **2018**, *8* (6), 3108–3119.

(12) Chen, J.; Sharapa, D.; Plessow, P. N. Stability and Formation of Hydroxylated α-Al_2O_3(0001) Surfaces at High Temperatures. *Phys. Rev. Res.* **2022**, *4* (1) 013232.

(13) Leiko, O.; Kosto, Y.; Mašek, K. Structural and Photoelectron Studies of Sn-SnO_x and SnO_2 Nanoparticles on TiO_2 (110) Surface. In *Surface and Interface Analysis*; *Surf. Interface Anal.* **2018**, 50, 1116–1121.
